# Aloe derived nanovesicle as a functional carrier for indocyanine green encapsulation and phototherapy

**DOI:** 10.1186/s12951-021-01195-7

**Published:** 2021-12-20

**Authors:** Lupeng Zeng, Huaying Wang, Wanhua Shi, Lingfan Chen, Tingting Chen, Guanyu Chen, Wenshen Wang, Jianming Lan, Zhihong Huang, Jing Zhang, Jinghua Chen

**Affiliations:** 1 The School of Pharmacy, Fujian Medical University, Fuzhou, 350122 Fujian People’s Republic of China; 2 Fujian Key Laboratory of Drug Target Discovery and Structural and Functional Research, The School of Pharmacy, Fujian Medical University, Fuzhou, 350122 Fujian People’s Republic of China; 3 Fujian Province New Drug Safety Evaluation Centre, Fujian Medical University, Fuzhou, 350122 Fujian People’s Republic of China; 4 Public Technology Service Center, Fujian Medical University, Fuzhou, 350122 Fujian People’s Republic of China; 5 Department of Chemical Biology, College of Life Sciences, Fujian Agriculture and Forestry University, Fuzhou, 350002 Fujian People’s Republic of China

**Keywords:** Aloe derived nanovesicles, Drug carriers, Stability, Indocyanine green, Cancer therapy

## Abstract

**Background:**

Cancer is one of the devastating diseases in the world. The development of nanocarrier provides a promising perspective for improving cancer therapeutic efficacy. However, the issues with potential toxicity, quantity production, and excessive costs limit their further applications in clinical practice.

**Results:**

Herein, we proposed a nanocarrier obtained from aloe with stability and leak-proofness. We isolated nanovesicles from the gel and rind of aloe (gADNVs and rADNVs) with higher quality and yield by controlling the final centrifugation time within 20 min, and modulating the viscosity at 2.98 mPa S and 1.57 mPa S respectively. The gADNVs showed great structure and storage stability, antioxidant and antidetergent capacity. They could be efficiently taken up by melanoma cells, and with no toxicity in vitro or in vivo. Indocyanine green (ICG) loaded in gADNVs (ICG/gADNVs) showed great stability in both heating system and in serum, and its retention rate exceeded 90% after 30 days stored in gADNVs. ICG/gADNVs stored 30 days could still effectively damage melanoma cells and inhibit melanoma growth, outperforming free ICG and ICG liposomes. Interestingly, gADNVs showed prominent penetrability to mice skin which might be beneficial to noninvasive transdermal administration.

**Conclusions:**

Our research was designed to simplify the preparation of drug carrier, and reduce production cost, which provided an alternative for the development of economic and safe drug delivery system.

**Graphical Abstract:**

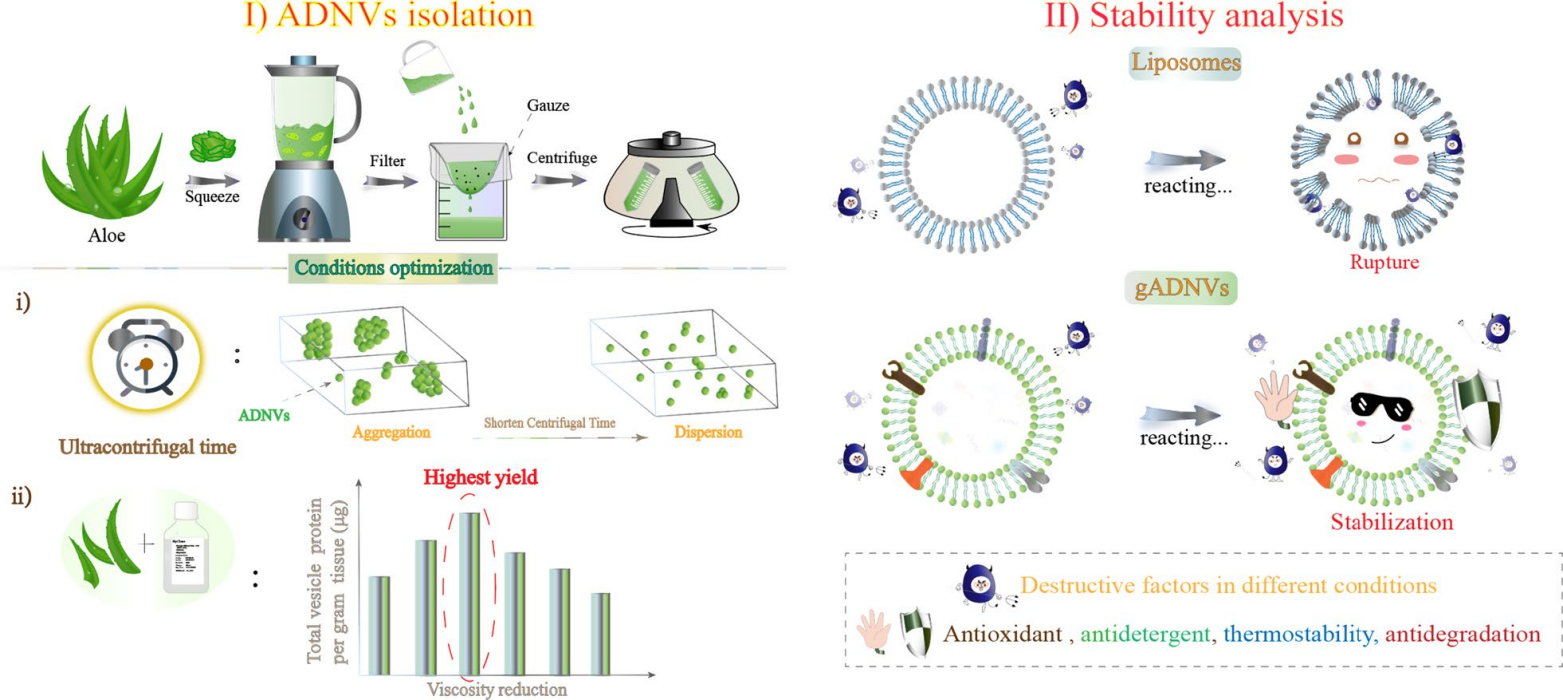

**Supplementary Information:**

The online version contains supplementary material available at 10.1186/s12951-021-01195-7.

## Background

Recently, phototherapies, such as photothermal and photodynamic therapy, have been emerging as powerful techniques for cancer therapy [1]. This type of therapy works only at the tumor site with both drug accumulation and penetrability near-infrared (NIR) laser irradiation, which effectively reduces drug resistance and the risk of toxicity to normal organ or tissue [2]. Currently, various photosensing materials have been proposed for photodynamic/photothermal synergistic cancer therapy [3]. Indocyanine green (ICG), as one of the U.S. Food and Drug Administration (FDA) approved drugs, has been widely used [4, 5]. However, free ICG is thermolabile, prone to bind to plasma proteins in blood, and easy to induce self-aggregation in an aqueous solution, limiting its delivery in vivo and application for cancer therapy [6]. Using nanocarriers for ICG encapsulation has been considered as a promising strategy to mitigate those problems. Currently, a great deal of nanocarriers in different forms have been reported for drug delivery, including liposomes [7], inorganic nanoparticles [8], nanocapsules [9], DNA nanomaterials [10, 11] and protein nanoparticles [6]. Therein, liposomes have been used clinically with permission [12]. Liposomes are lipid based spherical shaped vesicles with hydrophobic lipid bilayer and hydrophilic cavity [13]. The unique amphiphilic properties enable them to encapsulate wide types of lipophilic and hydrophilic drugs [14]. The advantages of liposomes lie in their flexible and controllable particle size, desirable biocompatibility and modifiability [15]. The capacity makes them interesting candidates as drug carriers. At present, liposomes have widely used for photosensitizer encapsulation and cancer therapy [16, 17]. However, the properties such as structure, charge and drug loading ability of liposomes are limited by lipid composition, which are hard to control independently [14]. The thermodynamic instability and oxidative degradation of phospholipids also hinder the further application of liposomes in the oncotherapy [18]. The addition of stabilizer or surface modification is a good way to improve the stability of liposomes, but with the increase of ingredients, more uncontrollable factors also increase [19]. Additional modification has been found leading to the vesicle deformation and increasing the risk of drug leakage [20]. Thus, the natural nanovesicle with great stability has its potential to be a better choice for drug delivery. Human extracellular vesicles (EVs) as living cell-secreted nanovesicles have been reported to have great potential in drug delivery [21]. Compared to artificial liposomes, EVs own natural lipid bilayer structure, which reduces the interference of multiple factors during vesicles preparation and is great for self-stability and prolonging the half-life in vivo. Regardless of the potential of EVs in drug delivery, the problems of high cost and low yield remain. Hence, an EV-like natural nanovesicle with the advantages of cost efficiency and high yield, is needed.

Fortunately, ongoing researches demonstrated that plants-derived nanovesicles (PDNVs) have similar properties to EVs [22]. PDNVs have various advantages, including sustainability, natural availability, renewability, and cost efficiency [23]. Unlike the isolation of human cell culture supernatants or body fluids derived EVs, PDNVs isolation do not require long-term cultivation or collection, which greatly reduces the preparation time [24]. It has been reported that PDNVs could communicate among interspecies without adverse effects. Grapefruit-derived nanovesicles (GDNVs) were found with no significant difference among the organs and pro-inflammatory cytokines of mouse after intervention, suggesting that GDNVs were safe to apply in vivo [23]. In addition, PDNVs are rich in various lipids and phytochemicals, bringing them unique biological characteristics. For instance, the ginger derived nanovesicles (GDNs) with abundant phosphatidic acid (PA) can be specifically attracted to intestine via the attraction of Lactobacillus rhamnosus to PA [25]. The special intestinal tendency endows GDNs great potential in intestinal administration. It is also worth noting that PDNVs can cross the blood–brain barrier (BBB) but not the placental barrier [26]. These properties allow PDNVs to be nanocarriers for drug delivery. At present, the research of PDNVs-based nanocarriers for drug delivery is still in infancy. Zhang et al*.* and Wang et al*.* used the lipid extracts of PDNVs to reconstruct new lipid nanocarriers, and studied their performances in detail [26, 27]. However, this recombinant method not only increased the complexity of carriers preparation, but also lost the functions of protein, nucleic acid or even phytochemicals contained in PDNVs, which did not fully reflected the properties of PDNVs as natural nanovesicles. Additional efforts to exploit the optimal approach for PDNVs isolation and analyze their capacities for drug delivery are needed.

**Scheme 1:**
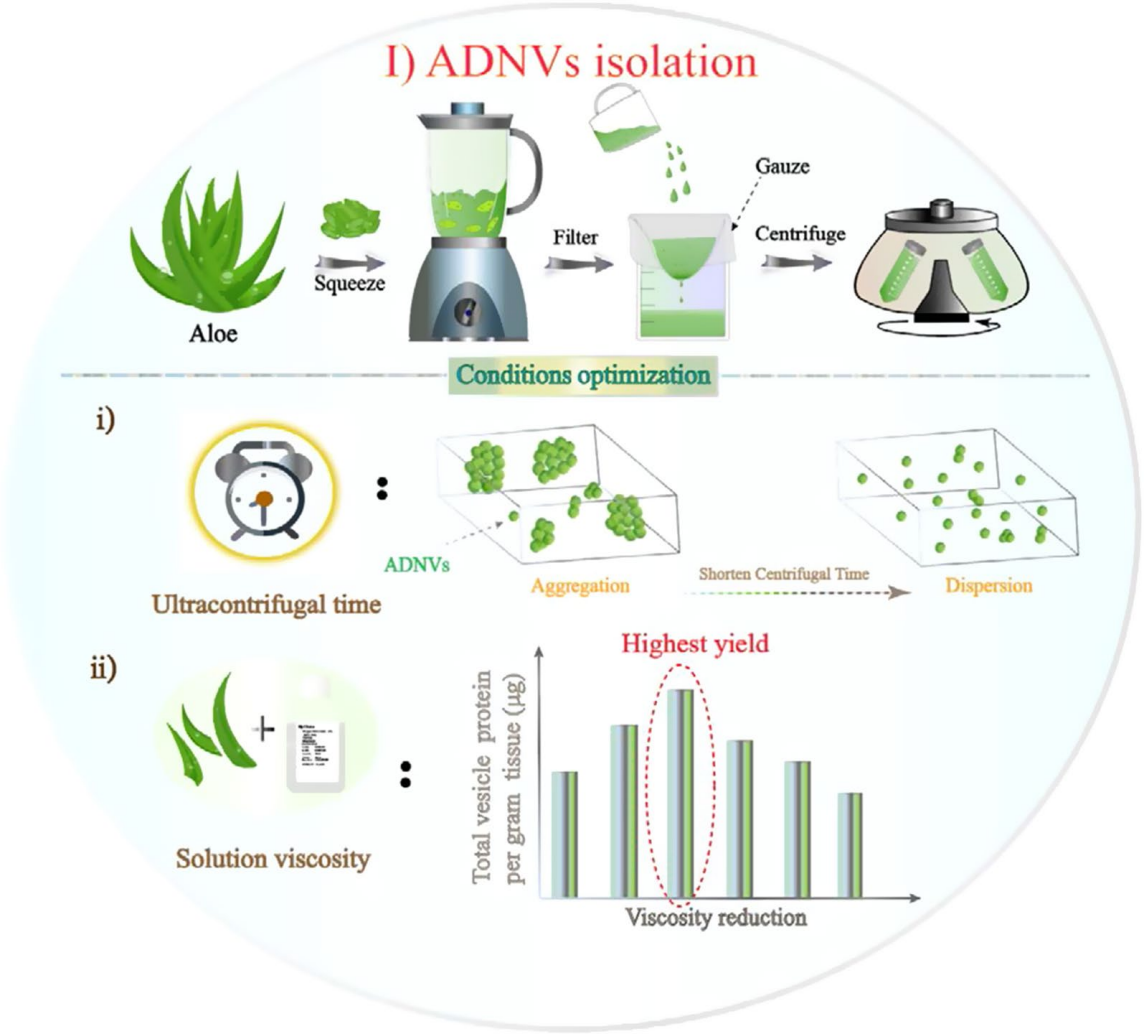
The isolation of ADNVs and conditions optimization

**Scheme 2:**
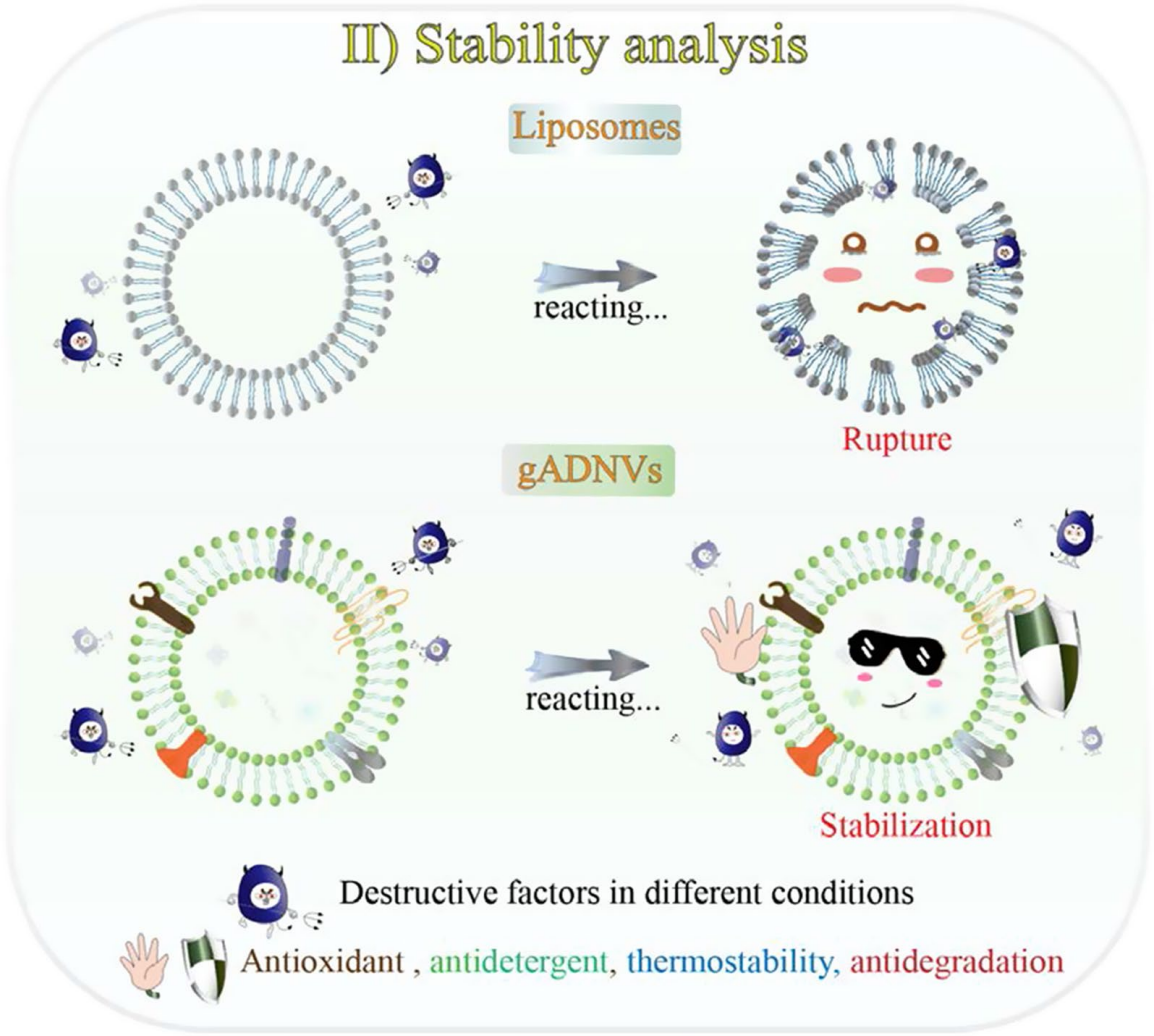
The stability analysis of ADNVs by reacting in different conditions

**Scheme 3:**
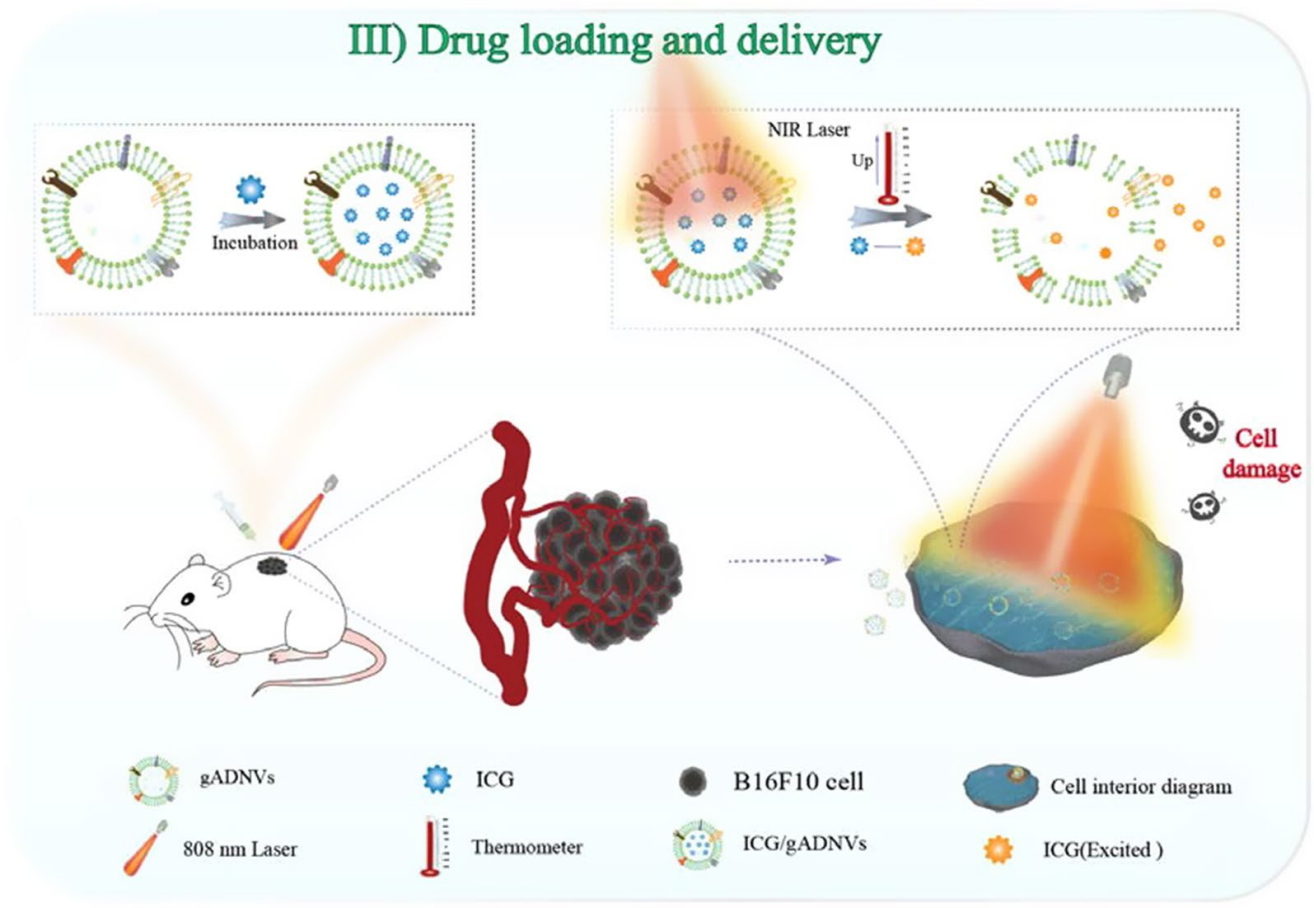
The ICG loading and delivery based on gADNVs for cancer therapy

Aloe as a mature medicinal herb has been reported with antioxidant, anti-inflammatory, anti-bacterial and other activities [28]. Currently, the nanovesicles derived from aloe (ADNVs) have been isolated and utilized in the studies of wound healing [29], NLRP3 inflammasome activity inhibition [30] and mitigating Insulin Resistance and Chronic Inflammation [31]. However, the current ADNVs isolation always used the similar method for EVs isolation. This was not necessarily the optimal method for ADNVs isolation. Herein, an optimized and simple method was proposed for aloe gel and rind derived nanovesicles isolating. We found that their particle size and yield were correlated to the final centrifugal time and the solution viscosity, respectively (Scheme 1). More monodispersed ADNVs could be obtained by shortening the centrifugal time, and adjusting the solution viscosity properly could improve their yield. The obtained ADNVs (including gADNVs and rADNVs) displayed EVs-like morphology, and both were slightly electronegative. Proteomic analysis showed that several proteins in ADNVs were the same as in reported PDNVs. Various lipids and some typical phytochemicals of aloe were also identified in ADNVs. The gADNVs with suitable particle size were used in the following study for drug delivery. We analyzed the potential of gADNVs as drug carriers from their structure and storage stability, antioxidant and antidetergent capacity, thermostability and stability in serum (Scheme 2). Their safety in vivo and in vitro was also investigated. In this system, ICG was efficiently loaded into gADNVs (ICG/gADNVs) and used for the melanoma therapy (Scheme 3). Our results showed that gADNVs had the protective and leakproof capacity for ICG. The cellular uptake, cytotoxicity and tumor therapy were effectively investigated via in vitro and in vivo experiments. Interestingly, the gADNVs showed prominent penetrability to mice skin which might be beneficial to noninvasive transdermal administration. This work indicated the pivotal factors of ADNVs isolation and provided detailed data of their components, which was beneficial to pave the way for the study of PDNVs in the future. The isolation of ADNVs was conducive to simplifying the preparation process of drug carrier, reducing production cost and improving drug storage time, which provided an alternative for the development of economic and safe drug delivery system and laid a foundation for exploring other plant derived nanovesicles for drug delivery and safe nanomedicine.

## Results

### Optimization of ADNVs fabricating condition

**Fig. 1:**
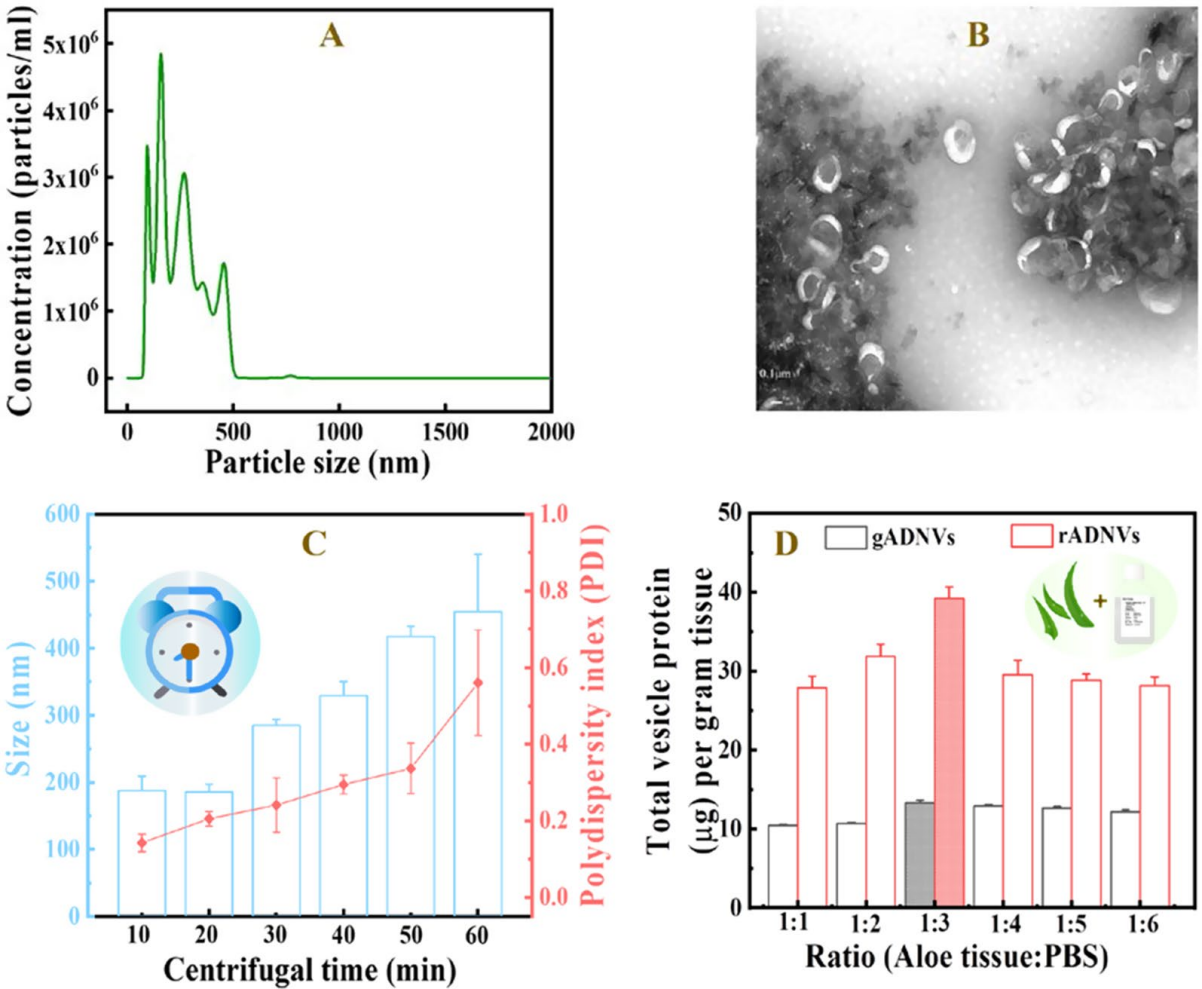
**A** The particle size of ADNVs isolated under high centrifugal force for 60 min analyzed by NTA. **B** The morphology and dispersion of ADNVs observed on TEM. **C** Evaluation of particle size and polydispersity (PDI) of ADNVs obtained with different centrifugation time. **D** The total vesicle protein (μg) per gram tissue isolated in different solution viscosities (the value on the bar chart). The color filled column (including red and gray) indicates the highest yield of gADNVs and rADNVs. Data in **C** and **D** represent mean values ± SD, n = 3

Currently, most of PDNVs were isolated by the method for EVs isolation under high centrifugal force for long centrifugation time [30, 32, 33]. The conditions were appropriate for the isolation of low amount of EVs in body fluid or cell culture supernatant [34, 35], however, the amount and the fluid environment of PDNVs were different from EVs. Thus, the method for EVs isolation was not assuredly suitable for PDNVs isolation. Mu et al*.* isolated the PDNVs from four edible plants using the similar method for EVs (ultra-centrifugated at 150,000×*g* for 90 min) [36]. Their results showed that the particle size of obtained PDNVs was uneven, some of which even up to 1000 nm. Also, Kim et al. produced many large vesicles by ultra-centrifuging at 100,000×*g* for 70 min when isolating ADNVs. To obtain smaller ADNVs, the filtration and ultrafiltration were used [29]. However, it was more complex, and may even reduce the yield of ADNVs, which was not conducive to mass production of vesicles. In our study, we found that the large vesicles might be caused by the vesicle aggregation (ultra-centrifugated at 100,000×*g* for 60 min). The micrograph of transmission electron microscopy (TEM) showed obvious agglomerations of ADNVs (Fig. 1B). The particle sizes of ADNVs ranged from 50 to 500 nm and were polydispersed (Fig. 1A). This kind of ADNVs was liable to be cleared by immune system and not suitable for in vivo delivery. To address this problem and obtain high quality of ADNVs to meet drug delivery needs, the isolating conditions were optimized. Given that the high centrifugal force (100,000–120,000×*g*) was more beneficial to isolate smaller EVs [35], we optimized the centrifugation time under the fixed centrifugal force of 100,000×*g*. The particle size and the polydispersity index (PDI) as the pivotal physical attributes of lipid-based nanocarriers [37] were monitored by dynamic light scattering (DLS) measurements (Fig. 1C). The results exhibited the average size of ADNVs isolated by 10 min and 20 min centrifugation were similar, and their PDI were 0.14 and 0.21 respectively. However, both the size and the PDI of ADNVs were evidently increased as the centrifugal time prolonged. With 60 min of centrifugation, the maximum size and PDI of ADNVs were up to 553.4 nm and 0.59, which indicated that the prolonged centrifugation time was the key factor leading to uneven particle size of ADNVs. Previous research reported that a PDI of 0.3 and below was acceptable in the drug delivery applications using phospholipid vesicles [38]. Meanwhile, we found the total protein of ADNVs was increased as a function of centrifugation time (Additional file 1: Fig. S2). According to the literature [39], the total protein of vesicles could be used as a quantitative index to measure the yield of EVs. Therefore, considering the yield and the quality of ADNVs, 20 min of centrifugation was selected for our subsequent study.

Since the aloe was rich in polysaccharide, resulting in a high viscosity, which might affect the isolation efficiency [40], the solution viscosity was modulated via adjusting the ratio of aloe juice and PBS. Meanwhile, given the content of polysaccharide was different in aloe rind and gel, we further isolated the ADNVs from aloe rind and gel respectively (denoted as rADNVs and gADNVs) (Fig. 1D). As the viscosity decreasing, the total amounts of proteins in gADNVs and rADNVs were both raised initially and then decreased, with the peak appeared at the viscosity of 2.98 mPa S and 1.57 mPa S respectively (Additional file 1: Table S1). This result indicated that the solution viscosity should not be ignored when comparing isolated ADNVs from aloe or PDNVs from other plants with similar property. Hereto, we concluded the primary factors for an efficient ADNVs isolation: (i) controlling the centrifugation time within 20 min; (ii) adjusting the viscosity of aloe juice for a higher yield.

### Characterization and component analysis of ADNVs

**Fig. 2:**
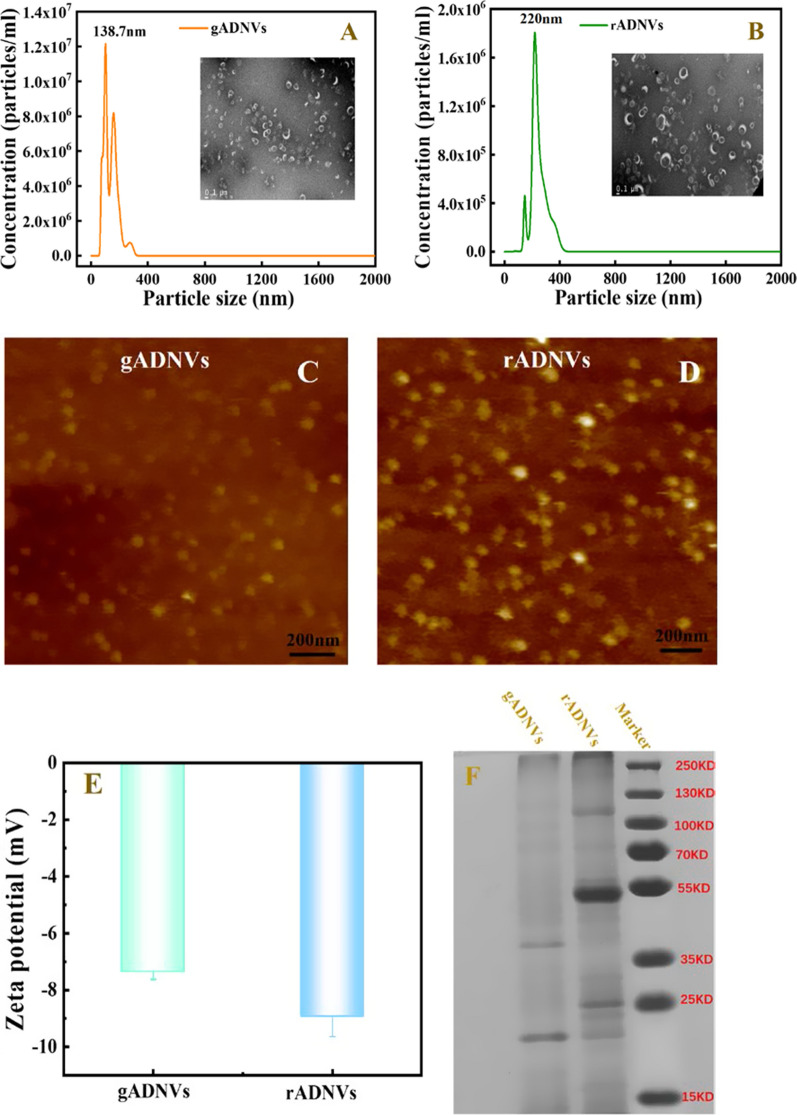

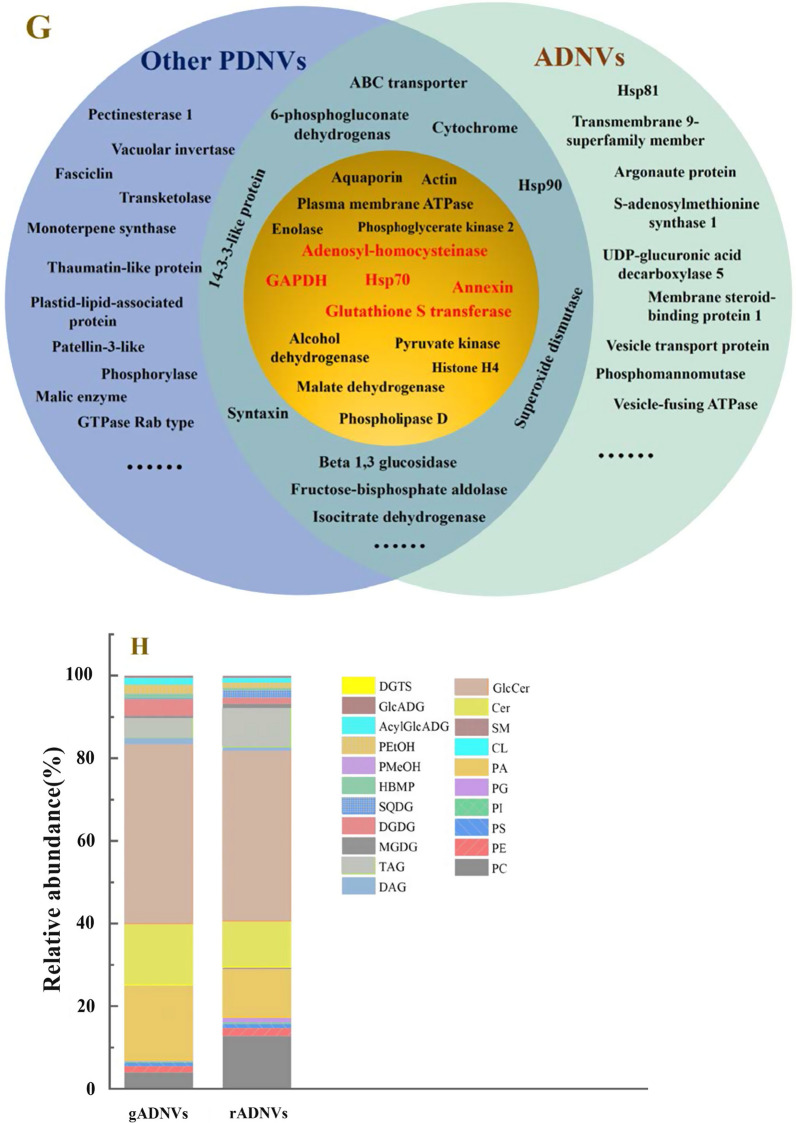
Characterizations of ADNVs obtained by the optimized method we proposed. **A**, **B** The particle sizes of gADNVs and rADNVs analyzed by NTA. Insets are their morphology and particle size distribution observed on TEM. **C**, **D** AFM micrographs of gADNVs and rADNVs. **E** The zeta potential analysis of gADNVs and rADNVs. **F** The protein distribution in two kinds of ADNVs analyzed by SDS-PAGE and stained by CBB (Coomassie brilliant blue). **G** The diagram showing the overlap of the proteins in ADNVs and other PDNVs. The proteins in the yellow circle were repeatedly identified in at least three kinds of PDNVs, while the proteins bold in red were repeatedly identified in at least five kinds of PDNVs. **H** The lipids compositions of gADNVs and rADNVs (the full name of lipids could be seen in Additional file 1: Table S4)

To fully analyze ADNVs obtained under optimal conditions, the properties (including particle size, morphology, zeta potential and protein distribution) of ANDVs were characterized. The particle size distribution range of ADNVs, deriving from aloe vera gel (Fig. 2A) or rind (Fig. 2B), was significantly reduced comparing to the aforementioned result in Fig. 1A, B. The TEM micrographs displayed the morphology of ANDVs with a distinct elliptic or saucer-like phospholipid bilayer. Also, some typical spherical particles were observed by atomic force microscopy (AFM) (Fig. 2C, D). It was worth noting that the size of ADNVs in AFM micrographs was smaller (< 100 nm) which might be due to the compression to vesicles caused by the surface tension of evaporating water [41]. As previous study reported, air-drying could severely alter the morphology of EVs and EVs may collapse and get flatten or shrunk to half of their normal volume when dehydrated [42, 43]. Additionally, better particle dispersion could be observed in both TEM and AFM results, indicating that the centrifugation with shorter time was better for ADNVs isolation. Under the optimized condition, the average particle size of gADNVs and rADNVs analyzed by nanoparticle tracking analysis (NTA) was 138.7 nm and 220 nm, respectively. The zeta potential of gADNVs and rADNVs was − 7.4 mV and − 8.9 mV respectively (Fig. 2E). To reduce protein adsorption, the nanoparticles (NPs) bearing slight negative charges were beneficial to drug delivery in vivo [44]. Thus, the gADNVs and rADNVs with good dispersion and slight negative charges may be suitable to be used as drug carriers.

Subsequently, the components (including proteins, lipids and phytochemicals) of two kinds ADNVs were characterized. A SDS-polyacrylamide gel electrophoresis (SDS-PAGE) was performed for total protein distribution assay. The protein from gADNVs and rADNVs showed different distributions in a range of 15–250 KD (Fig. 2F). Protein profile analysis displayed that various proteins presented in gADNVs and rADNVs, such as transmembrane proteins, cytoskeleton protein and RNA-binding protein (Additional file 1: Tables S2 and S3). As Fig. 2G (the intersection of two circles) showed, more than 25 types of proteins in ADNVs were the same as in reported PDNVs. Particularly, the heat shock protein (HSP 70), glutathione S-transferase, annexin families, adenosylhomocysteinase and glyceraldehyde-3-phosphate dehydrogenase (GAPDH) had been repeatedly identified in at least five kinds of PDNVs (the yellow circle in the middle) [45–49]. In addition, beta 1,3 glucosidase as a cell wall remodeling enzyme was also identified in ADNVs. As previously reported, the secretion of PDNVs through cell wall may be due to the remodel of cell wall by relative enzymes contained in PDNVs [50, 51], which provided evidence that ADNVs secretion was from cells of aloe. We also found that both gADNVs and rADNVs contained RNA-binding proteins, i.e. argonaute (AGO) protein. AGO proteins coupled with sRNAs to induce the silencing of genes with complementary sequences [52], which might relate to regulate the encapsulation of small RNA in ADNVs. These results suggested that ADNVs as functional vesicles might play an important role in the transportation of cargos in aloe.

Lipidomic analysis exhibited that various phosphatides were enriched in ADNVs. As Fig. 2H showed, glucosylceramide (GlcCer) was the primary lipid in both gADNVs and rADNVs (~ 43.55% and ~ 41.33% of total lipid, respectively), other lipids including ceramide (Cer, 14.98% and 11.24%, respectively), phosphatidic acid (PA, ~ 18.12% and 11.81% of total lipid, respectively) and phosphatidylcholine (PC, ~ 3.85% and ~ 12.77% of total lipid, respectively) also accounted for a large proportion. Previous research showed that GlcCer was usually regarded as a minor component with less than 5% of the total lipid extract of plant tissues, but about 6–30% of the total membrane lipids in the plasma and vacuolar membrane of plants [53]. The large proportion of GlcCer in both gADNVs and rADNVs might be due to the fusion between multivesicular bodies (MVBs) and the plasma membrane in vesicle secreting process [54]. Meanwhile, ceramide as a critical component with properties of facilitating or inducing membrane curvature in exosome formation and secretion was also found in both kinds of vesicles. These results may suggest the presence of the natural secretion of ADNVs. In addition, PA, being a lipid mediator, could modulate membrane fission and fusion and also influence PDNVs uptake by triggering cytoskeleton rearrangement [55]. There were also some other lipids contributing to lipid stability. For example, the PC with antioxidant capacity was in favor of reducing oxidative degradation of lipid [49], the digalactosyldiacylglycerol (DGDG) and monogalactosyldiacylglycerol (MGDG) could stabilize phospholipid during freeze-thawing and freeze-drying [56]. In general, these lipid components played an important role in explaining the origin and function of ADNVs.

Different from EVs, plant derived nanovesicles are rich in various homologous active ingredients. For example, shogaol and gingerol have been identified in ginger derived nanovesicles (GDNVs), which endowed GDNVs unique pharmacological activity [57]. The analysis of phytochemicals of parent plants may be regarded as an indicator for PDNVs identification. Aloe as a traditional herb has shown containing various pharmacologically active ingredients, a typical kind of which was anthraquinones, such as aloe-emodin and aloesin. To further analyze, we performed the high performance liquid chromatography (HPLC) to quantify the phytochemicals, including aloe-emodin, aloesin and β-sitosterol, in gADNVs and rADNVs respectively. As Additional file 1: Fig. S3 showed, all these phytochemicals were detected in both gADNVs and rANDVs. The contents of Aloe-emodin, aloesin and β-sitosterol in gADNVs and rADNVs were exhibited in Additional file 1: Table S5. Aloe-emodin and aloesin were more abundant in aloe rind, while β-sitosterol was more in gel. According to the previous studies, aloe-emodin, aloesin and β-sitosterol possessed various properties, and the antioxidant capacity was one of them [58–60]. As known, the phospholipid oxidative degradation was one of primary causes leading to vesicle instability [61], the antioxidative compositions can help with the ADNVs stability. In addition, β-sitosterol with the function of adjusting membrane fluidity promotes ordered phospholipid arrangement, which had been used as a stabilizer for improving the stability of liposome [62]. Therefore, the ADNVs with various active ingredients are better suitable for drug delivery.

### The feasibility of ADNVs for drug loading and delivery

According to aforementioned results, both of gADNVs and rADNVs were with slight negative charge, and their average sizes were 138.7 nm and 220 nm respectively. It has been reported previously that slight negative charge and particle size below 150 nm facilitate the internalization and accumulation into tumor [37]. Thus, gADNVs with size below 150 nm were selected as drug carrier for our subsequent study. As lipid nanocarriers, the morphology and attribute of gADNVs were similar to liposome. Thus, liposome can be a great reference for investigating the performances of gADNVs. Herein, PC and phosphatidyl ethanolamine (PE) as the common phospholipids for liposomes synthesization [63] were used as the primary ingredients. Given that gADNVs were rich in various phospholipids, another phospholipid, phosphatidyl glycerol (PG), was added to design a multicomponent liposome as the reference of gADNVs. As aforementioned, the presence of various components in gADNVs may allow gADNVs to be more stable. As a proof of concept, the reference liposomes, as the blank control, were prepared without adding stabilizer. The feasibility of gADNVs for drug loading and delivery was investigated as follows.

### Characterization of liposomes

The average particle size of Lips was 91.9 nm analyzed by NTA analysis, and the TEM micrograph showed monodispersed spherical shapes (Additional file 1: Fig. S4A). The structure of Lips was analyzed and compared with gADNVs by Fourier transform-infrared (FT-IR) spectroscopy (Additional file 1: Fig. S4B). The coexisting carbohydrate region and signature amide I band indicated the similar vesicle structure between gANDVs and Lips, which suggested that the synthesized liposomes were qualified as references for gADNVs.

### Stability and antioxidant capacities of gADNVs

**Fig. 3:**
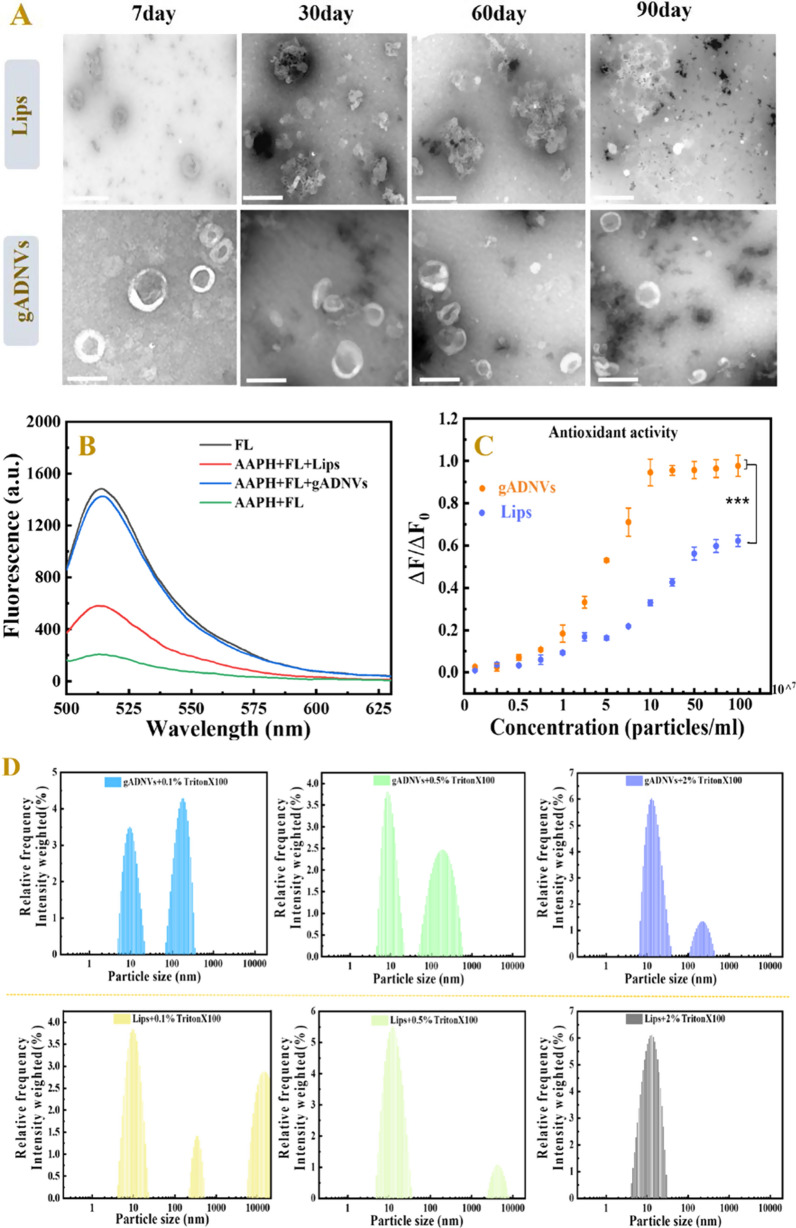
The stability of gADNVs and Lips. **A** TEM micrographs of two kinds of vesicles at different storage stages. **B** The recovery of FL fluorescence after interacting with gADNVs or Lips at a concentration of 1 × 10^8^ particles/mL. **C** The antioxidant capacity of gADNVs and Lips evaluated by ORAC. **D** Particles size characterization of gADNVs and Lips intereacted with TX-100 of different concentrations. Data in **C** represents mean values ± SD, n = 3. Statistical differences were analyzed by two-tailed student’s t-test. ***p < 0.001

The stability of a drug carrier is crucial as it can affect the drug retention and delivery in vivo. To evaluate the stability of gADNVs and compare it with Lips, their morphology was observed by TEM under different stages. As shown in Fig. 3A, the integrated structure of gADNVs could be observed from the 7th day until the 90th day, while the membranous structure of Lips became indistinct and fragmented as the storage time went by. This intuitively reflected the excellent stability of gADNVs.

The stability of lipid-based nanocarriers could be embodied in antioxidant and antidetergent capacity. Thus, to further investigate their stability, the antioxidant and antidetergent capacity of gADNVs and Lips were analyzed. The antioxidant capacities of gADNVs and Lips were evaluated by oxygen radical absorbance capacity assay (ORAC). ORAC is a rapid method for determining total antioxidant capacity (TAC) in biological samples [64]. The 2,2-azobis (2-amidino-propane) dihydrochloride (AAPH) as the peroxul radical generator can cause the oxidation of fluorescein disodium (FL) and result in the fluorescence quenching. Only if antioxidants were present would the generated peroxul radical be plundered and then inhibited the oxidation of FL. A typical concentration based fluorescent recovery was analyzed when interacting with gADNVs. The results showed approximately 100% recovery at the concentration of 1 × 10^8^ particles/mL for gADNVs and less than 40% recovery at the same concentration for Lips (Fig. 3B). Furthermore, it’s difficult to recover the quenching fluorescence adequately even at the concentration of 1 × 10^9^ particles/mL for Lips (Fig. 3C). The results manifested that gADNVs as natural vesicles with various antioxidant ingredients were more stabilized than Lips, without the assist of stabilizer.

The interaction between detergents and vesicles is in virtue of the insertion of detergents to lipid bilayer which lead to micelles formation and vesicles structure disintegration, also named as solubilization [65]. It is a common analytical method for structural stability of vesicles [66]. To investigate the structural stability of gADNVs and Lips, the nonionic surfactant Triton X-100 (TX-100) as a membrane breaker was used and interacted with 1 × 10^8^ particles/mL of gADNVs and Lips for 30 min. According to previous study, the unimodal size distribution of vesicles was changed to bimodal or multimodal size distributions in the solubilization process, and the size distributions of vesicles were widened or disappeared [67]. As in Fig. 3D, at the concentration of 0.1% TX-100, the unimodal size distribution of gADNVs was preserved, and the bimodal size distribution of Lips appeared. As the concentration of TX-100 increased, the peak of size distribution of gADNVs declined yet remained at 2% TX-100 concentration. Conversely, the size distribution of Lips disappeared thoroughly at 2% TX-100. We believed that the various phospholipids in gADNVs allow more ordered phospholipids arrangement and prevented the insertion of TX-100 molecules into lipid bilayers, which limited the formation of the vesicles-detergent mixed micelles, thereby alleviating the dissolution. It allowed gADNVs remaining stable in complex internal environment.

### Safety assessment of gADNVs in vitro and in vivo

**Fig. 4:**
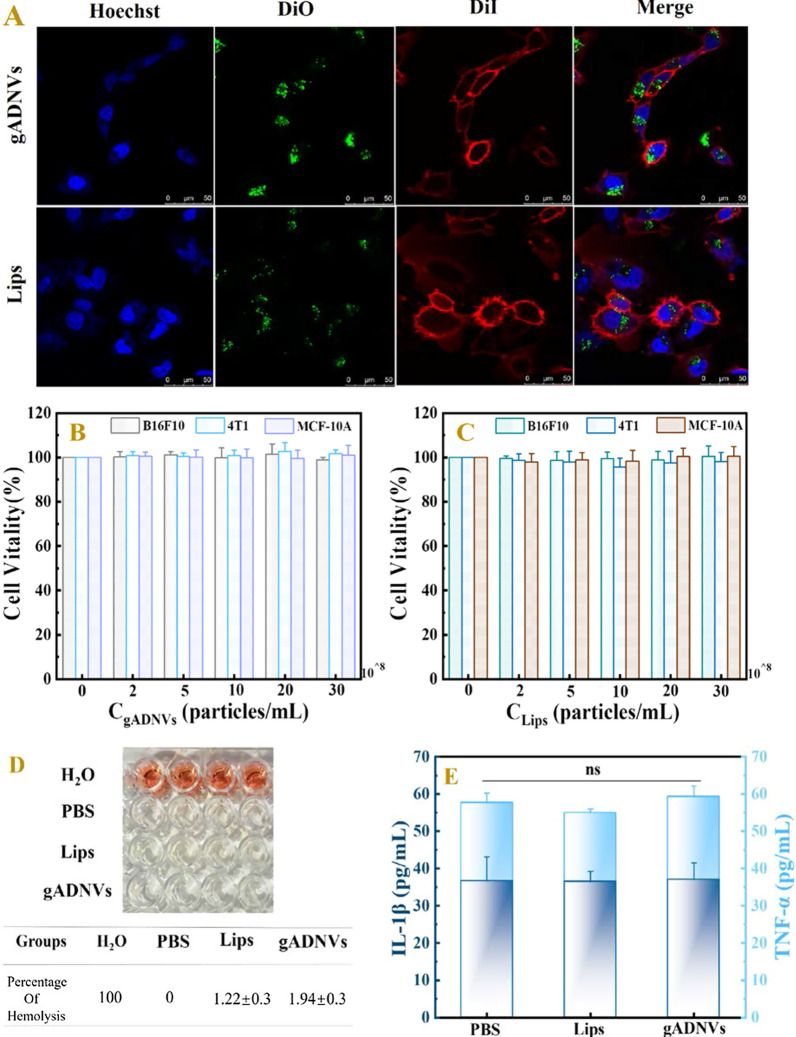

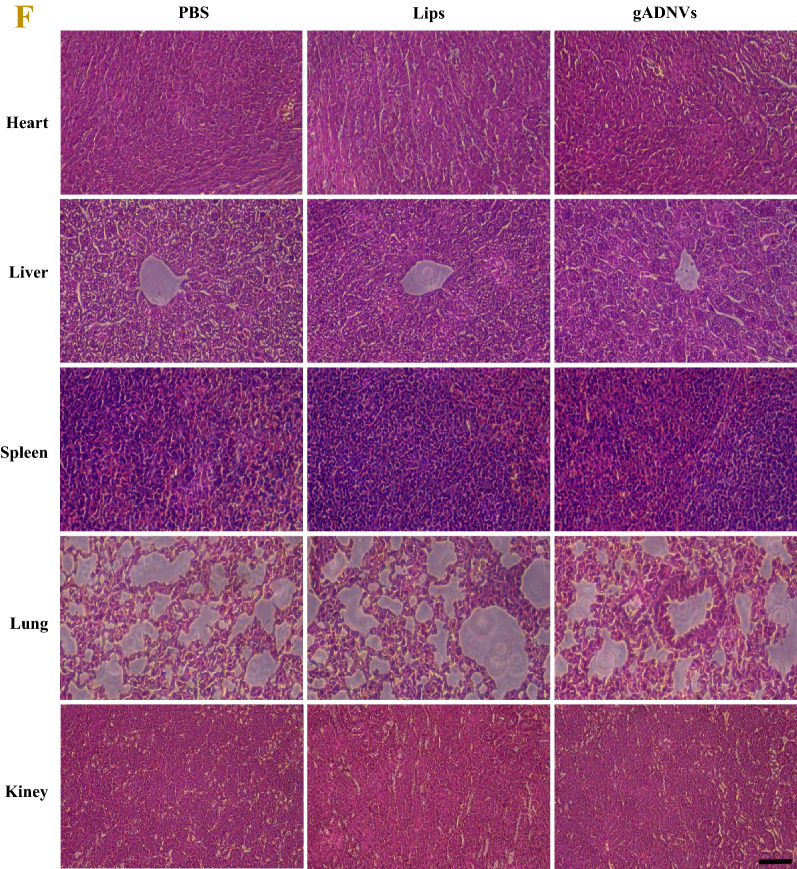
The safety assessment of gADNVs in vitro and in vivo. **A** The cellular uptake of gADNVs analyzed by confocal laser scanning microscope. DAPI (blue) was the nuclear staining channel; DiO (green) was the channel of gADNVs and Lips; DiI (red) was the cell membrane staining channel. **B** The vitality of B16F10, 4T1 and MCF-10A cells analyses by MTT assay after treating with different concentrations of gADNVs. **C** The viability of B16F10, 4T1 and MCF-10A cells analyzed by MTT assay after treating with different concentrations of Lips. **D** The hemolysis assay of gADNVs and Lips. The group treated with ultrapure water was set as a positive control with the hemoglobin release rate of 100%, and the group treated with PBS was used as a negative control. **E** The changes of proinflammatory cytokines (TNF-α, IL-1β) in mice after PBS, gADNVs and Lips injection. **F** H&E staining of heart, liver, spleen, lung and kidney sections treated with PBS, gADNVs and Lips. Scale bar was 100 μm. Data in **B**, **C** and **E** represent mean values ± SD, n = 3. Statistical differences were analyzed by two-tailed Student’s t-test, ns shows that there is no significant difference between the compared data

Cellular uptake is a crucial factor in drug delivery [68]. In this study, the green cell membrane dye (DiO) was used for gADNVs and Lips labeling (denoted as DiO/gADNVs and DiO/Lips), and for melanoma cell (B16F10) uptake study. As a cell membrane dye, free DiO generally tends to bind to the cell membrane rather than entering the cytoplasm [27]. As Fig. 4A showed, the punctate and green fluorescence of DiO was observed in cytoplasmic region rather than cell membrane, indicating that DiO in cytoplasm was derived from labeled gADNVs and Lips, which demonstrated the feasibility of gADNVs and Lips delivering drug into cells. On the premise of intake of gADNVs and Lips, their cytotoxicity was investigated for diverse cell lines by MTT assay. As Fig. 4B, C showed, both gADNVs and Lips had no obvious toxicity to B16F10, MCF-10A and 4T1 cells. The cell viability was not reduced with the increasing concentration of gADNVs and Lips, preliminarily suggesting that gADNVs and Lips were safe in vitro. Then, we investigated the potential in vivo toxicity of gADNVs by intravenous delivery. Firstly, the hemolytic test was preformed to assess the safety of gADNVs and Lips to erythrocyte. H_2_O and PBS was used as the positive and negative control respectively (Fig. 4D). The hemolysis rate of gADNVs and Lips were 1.22 ± 0.3% and 1.94 ± 0.3%, which is considered no hemolysis since the hemolysis rate is less than 5% [69]. It suggested that gADNVs were nontoxic to erythrocyte and could be used for intravenous administration. Next, the potential toxicity of gADNVs on systemic organs was explored. After intravenous injection with gADNVs or Lips for 7 days, the levels of proinflammatory cytokines in mice serum, such as interleukin (IL-1β) or tumor necrosis factor (TNF-α), did not change distinctly (Fig. 4E). The organ slices stained by histological analyses of hematoxylin–eosin (H&E) staining showed no evident damage on organ in either gADNVs or Lips treatment groups (Fig. 4F). Collectively, these results indicated that gADNVs could be taken up by melanoma cells and were safe in both vitro and vivo, and could be used as vectors for ICG to deliver for melanoma therapy.

### Effectively ICG loaded by using gADNVs (ICG/gADNVs)

Next, we investigated whether the well-stable gADNVs could be used for ICG loading. To obtain better loading efficiency, the optimum dosage of gADNVs was explored. Different mass ratios of ICG and gADNVs (the protein mass) were chosen during the preparation of ICG/gADNVs (Additional file 1: Fig. S5A). The loading efficiency tended to increase first and decrease afterwards, and a conspicuous peak showed at the mass ratio of 3:2, which indicated that the amount of gADNVs was important in drug loading. Meanwhile, to evaluate the performance of ICG/gADNVs, the ICG/Lips were prepared by thin film dispersion and hydration methods. The prepared ICG/gADNVs and ICG/Lips were characterized by DLS and TEM (Additional file 1: Fig. S5B, C). Their particle size were 129.8 nm and 101.3 nm, and zeta potential were − 8.1 mV and − 21.9 mV respectively (Additional file 1: Fig. S5D). These parameters met the requirements of drug delivery in vivo.

### Packaged ICG stability and leakage study

**Fig. 5:**
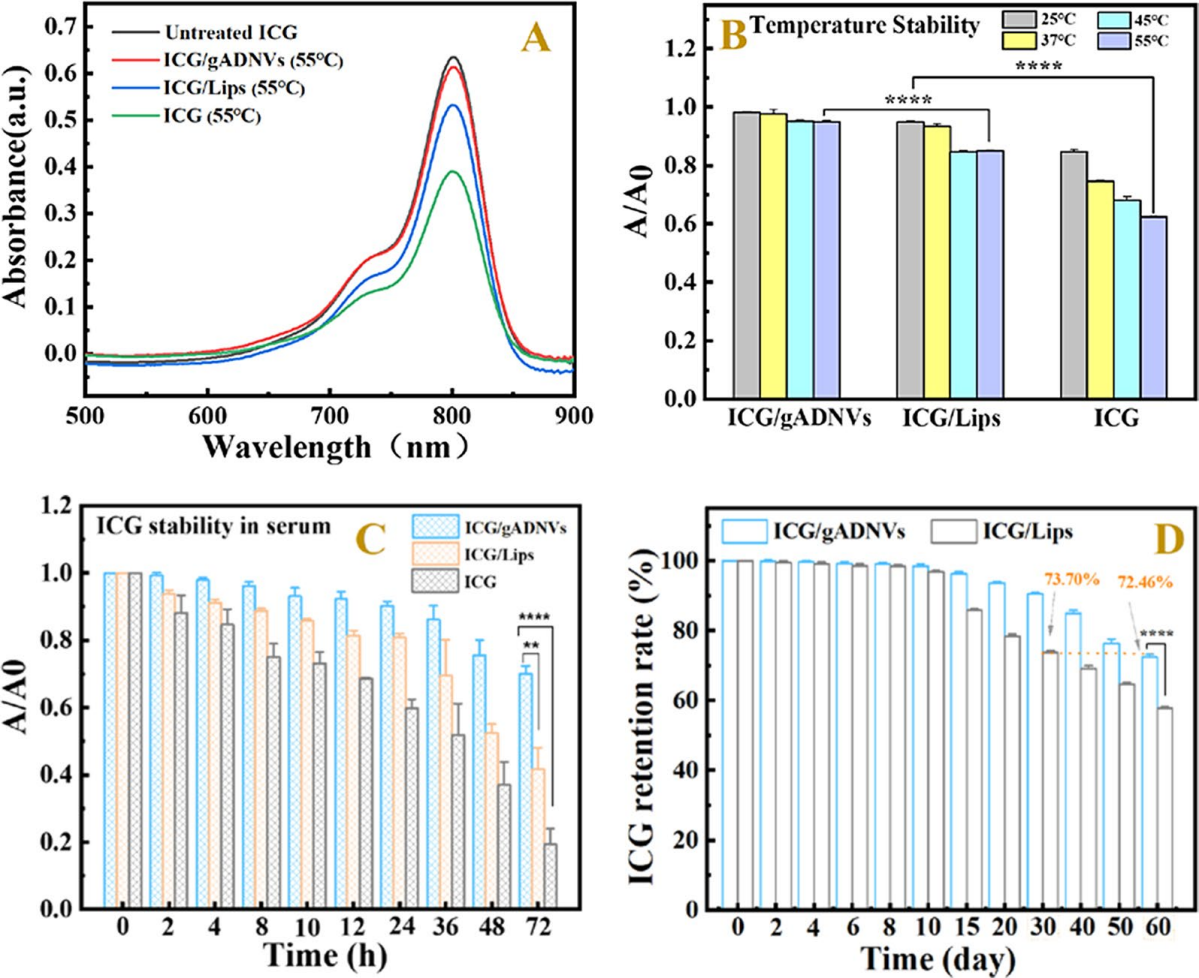
**A** The UV–vis spectra of ICG/gADNVs, ICG/Lips and ICG after 12 h incubation in 55 ℃ compared with untreated ICG. **B** The thermal stability of ICG after loading in gADNVs and Lips. A/A_0_ was the ratio of ICG absorbance before and after heating. **C** The stability of ICG/gADNVs, ICG/Lips and ICG in serum. **D** The retention rate of ICG in gADNVs and Lips after storage for different time. Data in **B**–**D** represent mean values ± SD, n = 3. Statistical differences were analyzed by two-tailed Student’s t-test. **p < 0.01, ****p < 0.0001

ICG was liable to bind to plasma proteins of blood and thus only had a transient blood residence time, and it was vulnerable to thermal degradation and hydrolysis due to the instability of methylene chains and imine cation [70]. Our results indicated that gADNVs was well-stable, which might protect drugs from degradation. To validate gADNVs as competent ICG carrier, the stability and leakage of ICG packaged in gADNVs were studied. The protective capacity of gADNVs to ICG was assessed by incubating in different temperatures and in serum for different periods of time. ICG/Lips and free ICG with the same concentration were prepared for comparison. The absorption of ICG was monitored as the evaluation indicator for the ICG thermostability comparison in PBS, Lips and gADNVs (Fig. 5A, B). There was distinctive difference of absorbance between ICG/gADNVs, ICG/Lips and free ICG. With the temperature increasing, only a slight absorption variation of ICG/gADNVs was observed, contrasting to a dramatic decrease of absorption of ICG/Lips or free ICG. The same trend was observed when in serum. The absorbance of ICG/gADNVs was significantly higher than that of ICG/Lips or free ICG (Fig. 5C). The results suggested that gADNVs had robust potency for ICG protection. Besides the protective effect on drug, preventing drug leakage was also a key to drug delivery. Thus, we subsequently investigated the leakproof performance of gADNVs for ICG via detecting the retention of ICG during long-term storage. As Fig. 5D displayed, there was more than 90% ICG retention in ICG/gADNVs after up to 30 days and 72.46% retention after 60 days storage. But it was only 73.70% ICG retention in ICG/Lips at 30th day, which demonstrated that gADNV was a favorable leakproof nanocarrier better than the Lips. These results showed that gADNVs had an excellent potential for drug loading, storage and delivery.

### Cytotoxicity assay

**Fig. 6:**
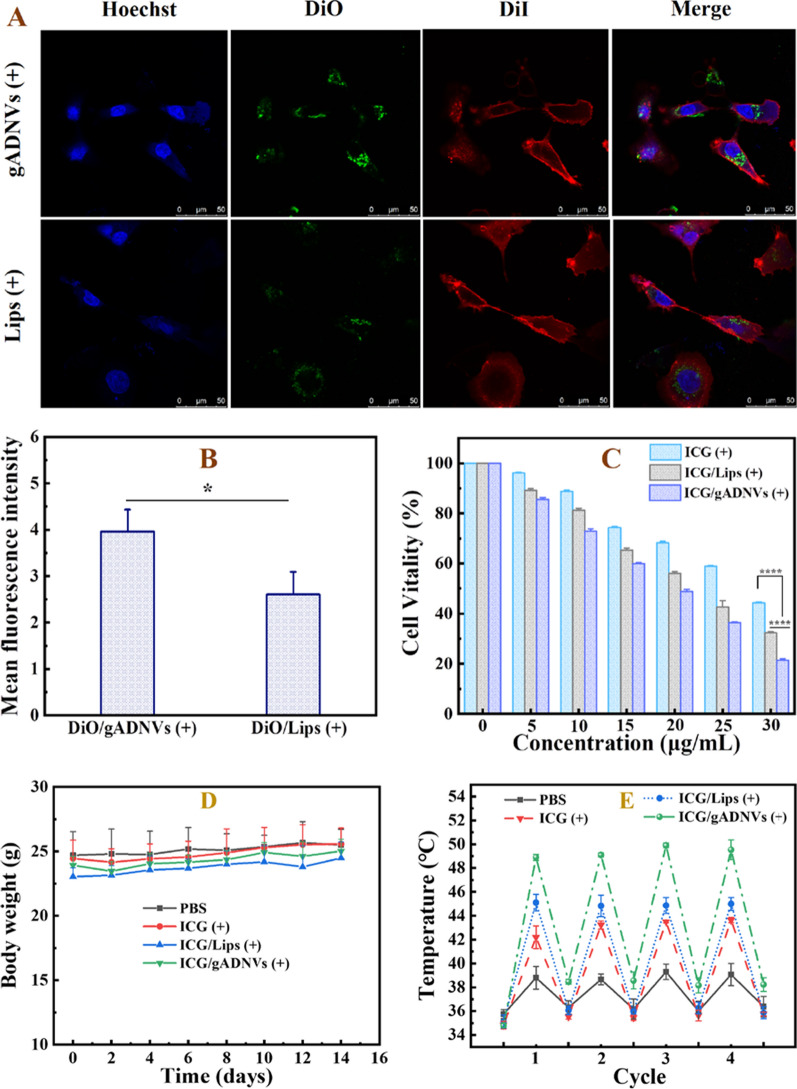

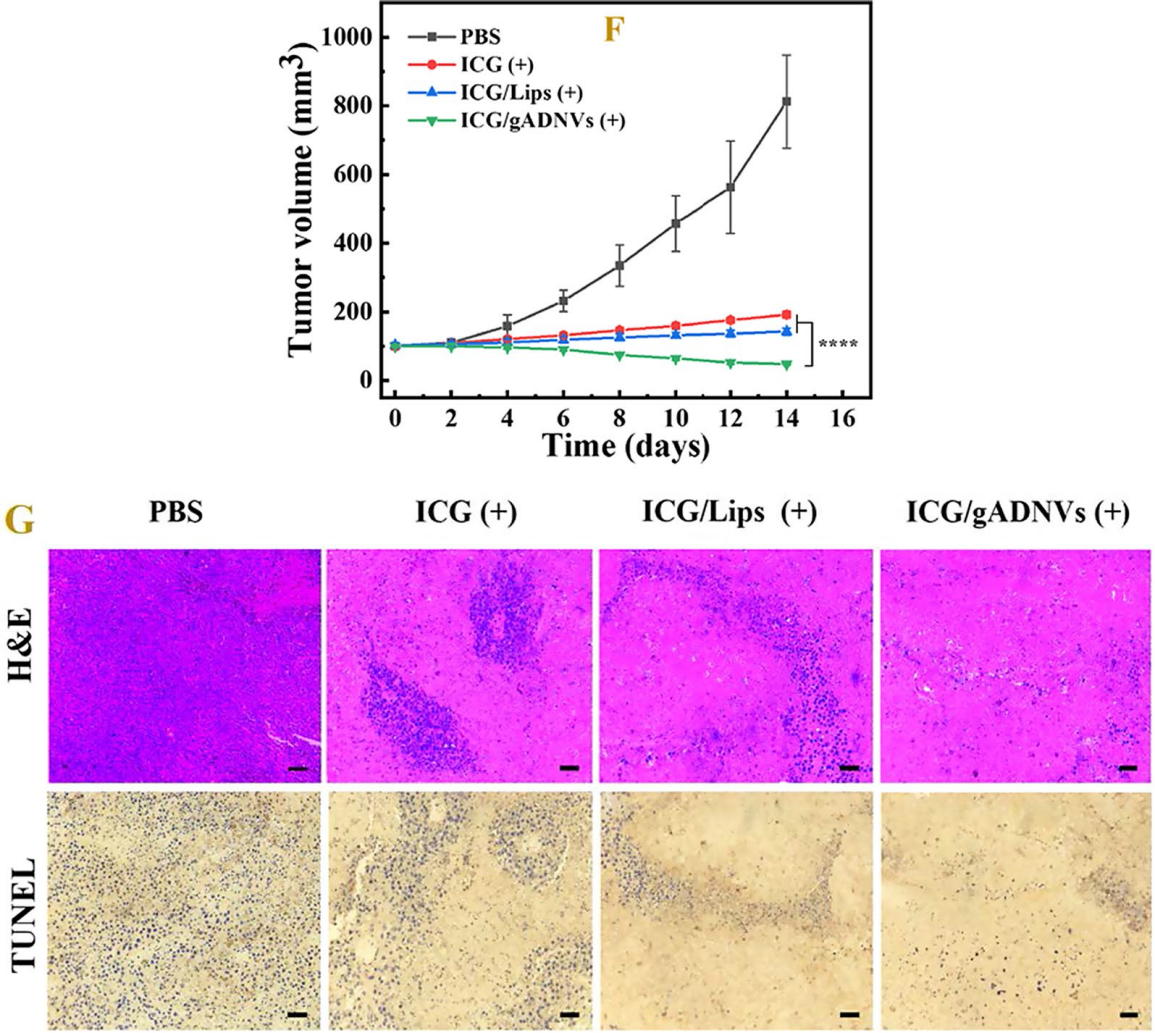
**A** Micrographs showing cellular uptake of DiO/gADNVs(+) and DiO/Lips(+) by confocal laser scanning microscope (CLSM). DAPI (blue) was the nuclear staining channel; DiO (green) was the channel of gADNVs(+) and Lips(+); DiI (red) was the cell membrane staining channel. **B** The mean fluorescence intensity of DiO/gADNVs(+) and DiO/Lips(+). **C** The cell damage effect of 30 days stored ICG(+), ICG/Lips(+) and ICG/gADNVs(+) after NIR laser irradiation. **D** Body weight changes of each group during phototherapy. **E** Temperature rising situation of each group after NIR irradiation. **F** Tumor volume changes of each group during therapy. **G** H&E staining and TUNEL staining of tumor tissue in each group. Scale bar is 50 μm. Data in (**B**) represent mean values ± SD, n = 3. Data in **D**–**F** represent mean values ± SD, n = 5. Statistical differences were analyzed by two-tailed Student’s t-test. *p < 0.05, ****p < 0.0001

As aforementioned that gADNV was a favorable leakproof nanocarrier better than the Lips, the cellular uptake of 30 days stored DiO/gADNVs(+) and DiO/Lips(+) were investigated by confocal laser scanning microscope (Fig. 6A). Notably, their intracellular fluorescence displayed distinct difference. The mean fluorescent intensity of DiO/Lips(+) was 1.34 times lower than DiO/gADNVs(+) (Fig. 6B). It demonstrated that gADNVs with excellent stability and drug retention were more conducive to drug delivery. Next, we explored the in vitro antitumor activity of ICG/gADNVs evaluated by MTT assay. The medicine groups were the 30 days stored ICG(+), ICG/Lips(+), and ICG/gADNVs(+) (Fig. 6C). The cytotoxicity exhibited dose dependence in each group. The result of cell damage showed the tendency of ICG(+) < ICG/Lips(+) < ICG/gADNVs(+) at each concentration. It might be due to the instability of ICG, and the leakage of ICG in ICG/Lips(+). These results exhibited that gADNVs as ICG carriers were beneficial to maintain the efficacy of ICG and may be better for ICG delivery in vivo.

### In vivo antitumor ability of ICG/gADNVs

**Fig. 7:**
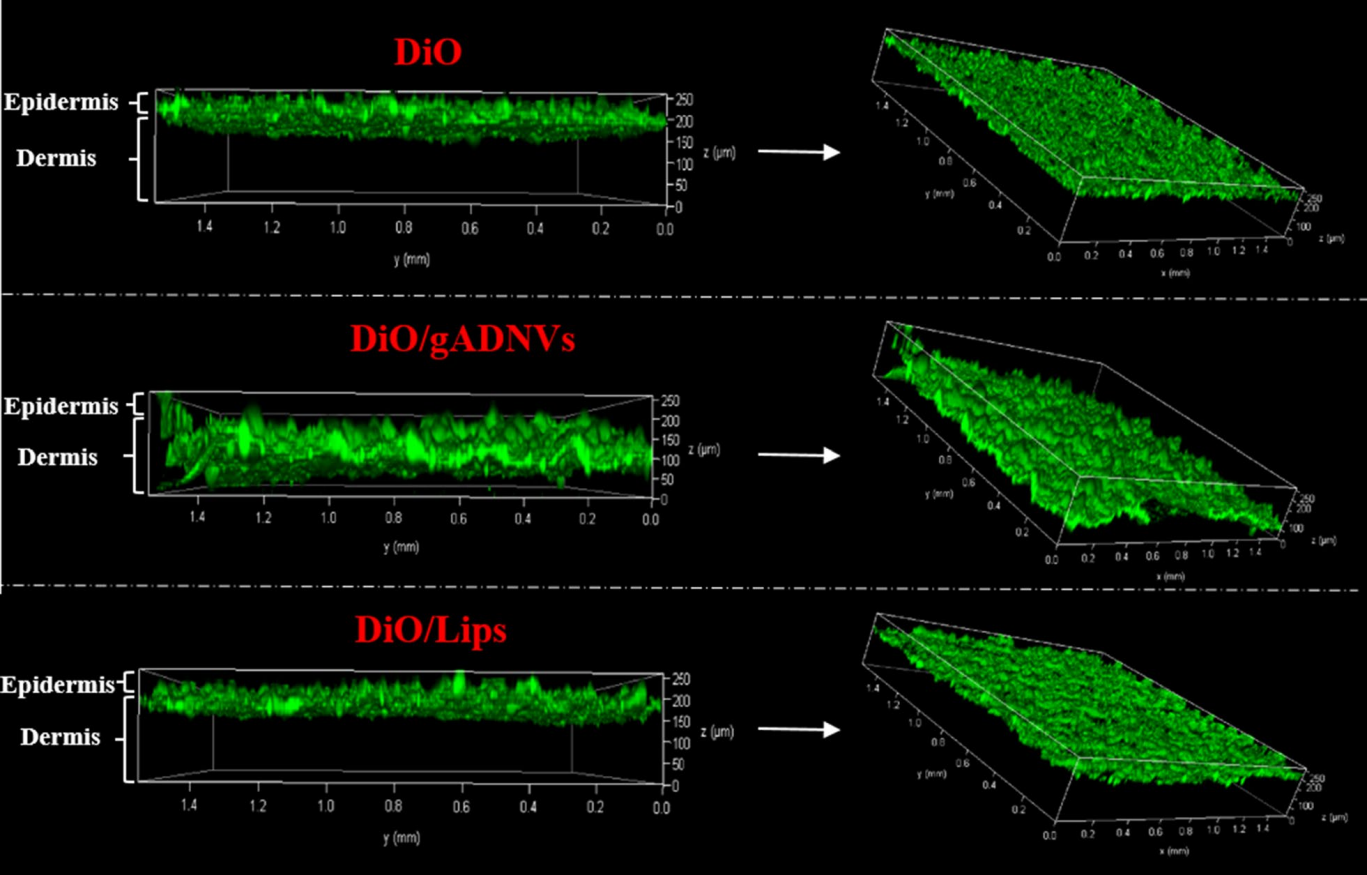
The penetrative situation of free DiO, DiO/gADNVs and DiO/Lips imaged by laser scanning confocal microscope

To evaluate the in vivo antitumor ability of 30 days stored ICG(+), ICG/Lips(+) and ICG/gADNVs(+), the bodyweight, temperature cycling, tumor volume, and histopathology were studied, with PBS being the control. The body weight of each group had no obvious change during therapy (Fig. 6D). After irradiated by NIR (808 nm, 1 W/cm^2^, 20 min), the temperature of each group showed varying degrees increased and retained 4 cycles (Fig. 6E). The PBS group at the temperature of 37.4 ℃ only increased about 4–5 ℃. The medicine groups showed the average temperature of 43.3 ℃ [ICG(+)], 44.9 ℃ [ICG/Lips(+)] and 49.4 ℃ [ICG/gADNVs(+)], which supported the efficacy of ICG was well retained in gADNVs during 30 days storage. As previous study showed, the temperature above 42 ℃ could cause cell damage, and the extent of cell damage was proportional to temperature [71]. The results were further reflected in changes of tumor volume (Fig. 6F). The inhibition of tumor volume in each group showed a trend of ICG(+) < ICG/Lips(+) < ICG/gADNVs(+). Furthermore, H&E staining and terminal deoxynucleotidyl transferase mediated nick end labeling (TUNEL) staining of B16F10 melanoma tumor tissue were performed (Fig. 6G). The tumor cells destruction of H&E and TUNEL-positive cells proportion also revealed a trend of ICG(+) < ICG/Lips(+) < ICG/gADNVs(+). We have also investigated the freshly prepared ICG, ICG/Lips and ICG/gADNVs containing the same amount of ICG for the anti-tumor efficacy study (Additional file 1: Fig. S6). The bodyweight of mice (Additional file 1: Fig. S6A) and the effect of tumor inhibition (Additional file 1: Fig. S6B, C) were similar as those of 30 days stored groups. There was barely any difference between the freshly prepared ICG/gADNVs and those after 30 days storage. However, the effect of ICG and ICG/Lips were significantly weakened after storage. The tumor growth of each group also displayed the similar trend (Additional file 1: Fig. S6D). These results manifested that ICG/gADNVs owned excellent tumor inhibited efficacy over an extended storage period, which was beneficial to reduce production and tumor therapy cost. It’s worth noting that gADNVs were found owning distinct transdermal performance via serial layer scanning on laser scanning confocal microscope. As the Fig. 7 showed, DiO/gADNVs penetrated the dermis and had a wide penetration range, whereas free DiO and DiO/Lips stayed in the epidermis mostly, which indicated that gADNVs as drug carriers may be used in skin cancer therapy by percutaneous administration.

## Discussion

In this study, we established and optimized the method for aloe rind and gel derived nanovesicles isolation and evaluated their applicability to ICG loading and melanoma therapy. The aloe gel derived nanovesicles were of uniform and suitable size, intact morphology and excellent stability, making them suitable for ICG loading and delivery.

In our initial study, we found that the universal method for EVs isolation was not the optimal condition for obtaining high quality of ADNVs, as obvious aggregation of vesicles could be observed after 60 min centrifugation. It had been reported that the wall effect was inevitable in centrifugation process, which might cause the vesicles to be squeezed, deformed and aggregated [72]. The extended centrifugation time aggravated this phenomenon. To obtain vesicles with higher quality, shortening centrifugal time to reduce the aggregation from wall effect on vesicles was necessary. To increase the yield of ADNVs in the optimized short time, the solution viscosity was taken into account. There has been reported that in the case of the same amount of EVs, the biological fluid with lower viscosity produced more EVs at the same centrifugation time [73]. Other studies also proposed that EVs isolation from biofluid (e.g. serum, plasma, saliva or milk) should be diluted at least 50% so to increase vesicles isolation efficiency [35]. Therefore, we measured the viscosity of aloe juice when the highest vesicles yield was obtained, and found that the optimal viscosity of the aloe gel and rind juice were different. This result suggested that, for the PDNVs isolation from different plants or plant tissues, the viscosity should be considered in order to achieve a high vesicle yield in a short time.

The ADNVs obtained under optimized conditions were characterized by several commonly used methods for EVs characterization recommended by MISEV2018 guidelines [74]. The vesicles isolated from aloe vera rind and gel showed similar EVs-like morphology, but other properties, including the average particle size, zeta potential, protein composition and distribution, lipid distribution, and content of phytochemicals, were different. Compared with rADNVs, the average particle size of gADNVs was more suitable for cancer therapeutic agents delivery. The results of proteomics showed that various transmembrane protein presented in ADNVs, which may be helpful for cell communication and benefit to drug delivery. Also, several proteins in both ADNVs were the same as in reported PDNVs. It is known that there is no reliable markers for PDNVs characterization currently [45]. The discovery of identical proteins in diverse PDNVs may be of great significance for the early establishment of a standard database of PDNVs markers.

As lipid nanocarriers, the properties of vesicles are closely related to their lipid composition. The lipids analysis showed that there were various lipids in ADNVs and rADNVs, including GlcCer, Cer, PA and PC, etc. Each of these lipids has diverse functional properties, which may endow ADNVs more bioactivities. In addition, the lipids in PDNVs also play other jobs. For instance, Sundaram et al*.* found ginger exosome-like nanoparticles (GELNs) were rich in phosphatidic acid (PA) and could be selectively taken up by the periodontal pathogen *Porphyromonas gingivalis* (*P. gingivalis*) in a PA dependent manner via interacting with hemin-binding protein 35 (HBP35) of *P. gingivalis* surface [75]. PA was also found in our obtained ADNVs, especially in gADNVs, which suggested that gADNVs may also has the same function. Also, GlcCer and Cer as the main components of skin lipids play an important role in the maintenance of water permeability barrier [76]. As our study exhibited, these two lipids composed 58.53% of the total lipids in gADNVs, making it easier to penetrate the cuticle barrier and reach dermis for drug delivery. Our study also verified that the gADNVs exhibited great transdermal property compared with liposomes and free dye, which was beneficial to construct a noninvasive agent for skin cancer (i.e. melanoma, in our study) treatment.

Additionally, phytochemicals as peculiar components in plants may be special markers for PDNVs identification. Herein, the typical aloe vera phytochemicals, aloe-emodin and aloesin, were analyzed by HPLC. We found that both of gADNVs and rADNVs contained aloe-emodin and aloesin. And they may be used as markers for the characterization of aloe vera derived nanovesicles due to their uniqueness in aloe. There has been reported that aloe vera gel contains no anthraquinones [77], which was inconsistent with our results. This may result from the communication of ADNVs in aloe vera, making the ADNVs in aloe vera gel mixed with the ingredients in rind. PDNVs have been verified participating in cargo transport between plant cells [78], and such behavior of transmission may be more conducive to inter-species communication.

## Conclusion

In summary, we proposed an optimized method for ADNVs isolation from aloe. In the study, we elucidated that the final centrifugation time was crucial when isolating aloe derived nanovesicles, and the solution viscosity was an important factor for obtaining higher vesicles yield. These findings might also be applied in other PDNVs isolation, providing an efficient way for obtaining high quality PDNVs. The obtained ADNVs were analyzed in morphology, particle size, zeta potential, proteins and lipids compositions, and phytochemicals contents, which was in favor of establishing a reliable database for PDNVs characteristics. The gADNVs isolated from aloe vera gel with suitable size, good stability and in vitro and in vivo safety were used for ICG loading and melanoma therapy. It showed a superiority than liposomes by simplifying the preparation process of drug carrier, reducing production cost and improving drug storage time, which provided a better alternative for the development of economic and safe ICG delivery system. Additionally, the transdermal performance of gADNVs showed the potential for noninvasive transdermal administration and skin cancer therapy.

## Materials and methods

### Isolation of ADNVs

Aloe was purchased in the local flower market. Firstly, 80 g aloe was washed five times with distilled water and peeled after. The gel and rind were then minced into pieces, mixed with ice-cold phosphate buffer solution (PBS, Hyclone) and squeezed by a kitchen blender. Subsequently, the filtration and the differential centrifugation were used to separate tissue residues and cell debris [79]. Briefly, aloe juice was filtered and centrifuged at 1000×*g* for 10 min, 3000×*g* for 20 min and 10,000×*g* for 45 min. Then, the final supernatant was centrifuged at 100,000×*g* for 10, 20, 30, 40, 50, and 60 min respectively. The preliminary pellets were washed twice and resuspended in ice-cold PBS. All the obtained suspensions were filtered by G-25 desalting column (CommaXP™, Biocomma Biotech Co., Ltd.), and the total proteins were measured by Bicinchoninic acid (BCA) kit and stored in – 20 ℃ for subsequent characterization.

### Characterization of ADNVs

The number and the particle size of ADNVs were characterized by NTA and DLS. Briefly, ADNVs were diluted with PBS to an appropriate concentration and injected into NanoSight NS300 instrument (Malvern Panalytical, Malvern, UK) to obtain 20–40 particles/view. Then, 60 s video was recorded at the temperature of 25 ℃. The data were analyzed on NTA 2.1 analytical software. For DLS measurements, a Litesizer 500 instrument (Anton Paar, Austria) equipped with 35 mW laser diode light (λ = 658 nm) was used for measuring the hydrodynamic size of ADNVs. The scattered light was collected at the angle of 90°. The analyzed samples of ADNVs were prepared with PBS at a ratio of 1:1000 and measured in light scattering at a fixed position.

To morphologically observe the isolated ADNVs, the micrographs by AFM and TEM were taken. For AFM analysis, specimens were diluted at a ratio of 1:10 with 2% paraformaldehyde and absorbed onto freshly cleaved mica sheets for 5 min. The surface morphology was surveyed by a Mulitmode 8 microscope (Bruker, USA). For TEM, ADNVs was mixed with PBS at pH 7.0 and vortexed thoroughly. To reduce observation interference, copper mesh inverted method was preferred. Twenty microliters of diluent was dropped onto a sealing membrane and covered with copper mesh (200-mesh carbon-coated, Beijing Zhongjingkeyi Technology Co., Ltd.) for 15 min. The copper mesh with ANDVs absorbed was then transferred onto a 2% paraformaldehyde droplet for 10 min. Lastly, 1% uranium dioxide acetate was used for 5 min for negative staining. The samples were observed under a Tecnai G2 microscope (FEI, U.S.A.). Unless otherwise stated, all measurements were operated at room temperature.

### Proteomic analysis of ADNVs

For proteomic analysis of ADNVs, ADNVs pellets were resuspended in lysis buffer consisting of 0.1% RapiGest SF Surfactant, 1% protease cocktail, and 50 mM NH_4_HCO_3_ (pH 8.0). The suspension was maintained at 90 ℃ for 20 min for thermal denaturation. Subsequently, the samples were reduced in 10 mM dithiothreitol (DTT) at 56 ℃ for 1 h, followed by alkylation of thiol group in darkness in 20 mM iodoacetamid (IAA) for 30 min at room temperature. Afterward, protein digestion was performed with a trypsin/protein ratio (m/m) of 1:50 at 37 ℃ for 18 h. To remove the RapiGest SF Surfactant, the digested protein sample was acidified by formic acid with a final concentration of 1%. After incubating at 37 ℃ for 30 min, the sample was centrifuged at 13,000 rpm for 10 min, and the supernatant was retrieved. The protein digest was analyzed by nanoflow RPLC-electrospray ionization tandem mass spectrometry (nano-RPLC-ESI–MS/MS) with an orbitrap FusionTM LumosTM TribridTM mass spectrometer coupled to an EASYnLC 1200 (Thermo Fisher, Waltham, MA, USA). The raw data were searched against the plant protein database downloaded from Uniprot (21, 620 entries) using Proteome Discoverer (version 2.3, Thermo Fisher Scientific).

### Lipidomic analysis of ADNVs

To analyze the lipid composition of ADNVs, total lipids of ADNVs were extracted firstly. In brief, 100 µL of ADNVs was extracted by mixed (0.75 mL) with methanol and methyl tertiary butyl ether (MTBE, 2.5 mL) for 1 h incubation at room temperature, and then added MS-grade water for phase separation. The upper phase was collected and dried. After that, the extract was dissolved in 100 µL of isopropanol and analyzed by UHPLC–MS/MS using Vanquish UHPLC system (Thermo Fisher, Germany) coupled with an Orbitrap Q ExactiveTM HF mass spectrometer (Thermo Fisher, Germany). Data were matched with the Lipidmaps, Lipidblast and HMDB database to obtain the qualitative and semi-quantitative results.

### Phytochemicals analysis of ADNVs

The aloe emodin, aloesin and β-sitosterol of ADNVs were analyzed by HPLC. The precipitations of ADNVs with 1 mg total protein were suspended in methanol and ultrapure-water (1:1 and 1:9 for aloe emodin and aloesin analysis respectively, v/v). The suspensions were vortexed and processed by sonication for 20 min, then centrifuged at 11,000 rpm for 5 min. The supernatants were collected and filtrated through 0.22 μm filters before analysis. For β-sitosterol analysis, 1:1 mixed ethanol aqueous solution and acetonitrile (v/v, 13:87) was used as extracting solvent to dissolve 0.1 mg ADNVs and processed by the same method mentioned above. The mobile phase conditions of HPLC were consistent with the corresponding extraction solvent of each component. The standard curve and the sample contents of aloe emodin, aloesin and β-sitosterol were analyzed at 254 nm, 254 nm and 205 nm [80] respectively, with a flow velocity of 1 mL/min. The column was maintained at 25 °C, 25 °C and 30 °C in the column oven respectively. Chromatography was performed on a LC-15C (Shimadzu corporation) system using Sepax BR-C18 column (4.6 mm × 250 mm, 5 µm).

### Stability and antioxidant of gADNVs

For the storage stability assay, multicomponent liposomes (Lips) were prepared as reference as shown in Additional file 1: Method and Materials S2. The long term stored gADNVs and Lips were observed at 7, 15, 30, 60 and 90th day at the stored temperature of – 20 ℃. The morphological variations corresponding to the aforementioned stored stage of vesicles were demonstrated by negative staining TEM micrographs. The antioxidant activity of gADNVs and Lips were assessed by Oxygen radical absorbance capacity assay (ORAC). In this method, 2,2-azobis (2-amidinopropane) dihydrochloride (AAPH, 1 mM) as free radical generating azo initiator interacted with substrate, fluorescein disodium (50 nM). Two kinds of samples with different concentrations reacted with above hybrid solution at 40 ℃ for 30 min. The fluorescent signals were monitored at the excitation wavelength of 480 nm by a fluorescent spectrophotometer (Hitachi, Japan) employing a micro quartz cuvette. To explore the structural stability of gADNVs and Lips, an analysis of resistance to detergent was performed. Different percentages of concentrations of Triton X-100 (TX-100) to vesicles were examined and actual size variations were recorded by DLS measurements.

### Preparation of ICG/gADNVs and ICG/Lips

For ICG/gADNVs preparation, ICG and gADNVs were incubated collectively. In brief, 300 μg/mL ICG was mixed with 200 μg/mL gADNVs (1 mL) and incubated at 37 ℃ for 30 min. The ICG/Lips was prepared according to Additional file 1: Method and Materials S2, except that the hydration solution was changed to ICG (300 μg/mL) solution dissolved in PBS. After that, two kinds of solutions were filtered by a G-25 desalting column to remove the unencapsulated ICG. Triton X-100 (TX-100, 10%) as a solubilizer was mixed into filtered solutions to dissolve two kinds of vesicles and released the packaged ICG for the calculation of ICG encapsulated efficiency.

$$ {\text{Encapsulated}}\, {\text{efficiency}}\,\left( \% \right) = \left[ {\left( {{\text{A}}_{{{\text{filtered}}{\kern 1pt} {\text{ICG}}{\kern 1pt} {\text{in}}{\kern 1pt} {\text{vesicles}}}} /{\text{A}}_{{{\text{unfiltered}}{\kern 1pt} {\text{ICG}}{\kern 1pt} {\text{in}}{\kern 1pt} {\text{whole}}{\kern 1pt} {\text{solution}}}} } \right)} \right] \times 100 $$

where A indicates absorbance of ICG in 800 nm.

### Packaged ICG stability and leakage study

The thermostability of ICG loaded in gADNVs was evaluated at 25, 37, 45 and 55 ℃ respectively, and compared with liposomal ICG and free ICG. Briefly, free ICG, ICG/gADNVs and ICG/Lips (10 μg/mL, concentration of ICG) were incubated in the metal bath at different temperature for 12 h. Then, 10% TX-100 was used to release ICG from vesicles thoroughly. The series of solutions were measured by a Cary Series UV–Vis Spectrophotometer (Agilent Technologies) and the relative absorbance (A/A_0_, absorbance after reaction/without reaction) of different samples were recorded.

To evaluate the stability of ICG/gADNVs in serum, free ICG, ICG/gADNVs and ICG/Lips (10 μg/mL, concentration of ICG) were incubated in 70% fetal bovine serum (FBS) at 37 ℃ for different periods of time. The absorbance of ICG was detected by a multimode reader (BioTek Instruments, Inc.). The stability of ICG was evaluated by recording the change of relative absorbances (A/A_0_, absorbance after reaction/without reaction) of different samples.

The leakage of ICG was evaluated by a long-term study of ICG/gADNVs and ICG/Lips at 4 ℃. Prepared ICG/gADNVs and ICG/Lips solution were stored at 4 ℃ for 0, 2, 4, 6, 8, 10, 15, 20, 30, 40, 50, and 60 days respectively. At each corresponding day, the solutions of ICG/gADNVs and ICG/Lips were filtered by G-25 desalting column to remove the leaked ICG. Filtrates were fixed to a certain volume by 10% Triton X-100. Then, the absorbance of ICG in each solution were measured. Each experiment was repeated three times in each group. The retentions of ICG in gADNVs and Lips were calculated by the following formula:

$$ {\text{Retention of ICG }}\left( \% \right) = \left( {{\text{A}}_{{{\text{ICG}}/{\text{gADNVs or ICG}}/{\text{Lips}}({\text{n}} = {\text{i}})}} /{\text{A}}_{{{\text{ICG}}/{\text{gADNVs or ICG}}/{\text{Lips}}({\text{n}} = 0)}} } \right)\, \times \,{1}00\% ,{\text{ i}} = 0,{ 2},{ 4},{ 6},{ 8},{ 1}0,{ 15},{ 2}0,{ 3}0,{ 4}0,{ 5}0,{ 6}0 $$

where A indicates absorbance of ICG in 800 nm, i is the corresponding storage time.

### Cell and animal

Murine melanoma cell line B16F10 cells, 4T1 cells and MCF-10A cells were provided by Cell Bank, Chinese Academy of Sciences. B16F10 cells and 4T1 cells were cultured in Dulbecco’s Modified Eagle Medium (DMEM, Gibico) and Roswell Park Memorial Institute (RPMI, Hyclone) with 10% fetal bovine serum (FBS), 1% penicillin and streptomycin (Thermo Fisher Biochemical Products (Beijing) Co., Ltd.). MCF-10A cells were cultured in Mammary Epithelial Basal Medium (MEBM) with 100 ng/mL cholera toxin. Cells were cultivated in a humidified atmosphere containing 5% CO_2_ at 37 ℃. Male BALB/c mice (20–25 g) were purchased from Shanghai SLAC Laboratory Animal Co., Ltd. All animal experiments were approved by the Laboratory Animal Management Committee of the Fujian Medical University (China, Approval number: FJMUIACUC2020-0112).

### Cellular uptake of gADNVs

Fluorescent labeling and confocal laser imaging were used for the analysis of cellular uptake. The gADNVs were first stained with DiO dye under 37 ℃ incubation. B16F10 cells were seeded in confocal petri dish (1 × 10^5^ cells/well) until adhered. After that, the solution of DiO/gADNVs (10 μg/mL of DiO concentration) containing medium was added for cell culture. Cells were washed thrice with PBS after 12 h incubation and fixed with 2% paraformaldehyde for 15 min to maintain cells morphology. Subsequent nuclear staining was performed via incubating cells with DAPI (10 μg/mL) for 15 min. Then cells were washed thrice again with PBS. A red cell membrane dye, DiI, was added to stain the cell membrane for a clearer cell profile. After 15 min culturing and thrice washing, the stained cells were stored in a sterile PBS solution and imaged on a TCS SP8 confocal laser scanning microscope (Leica, GER).

### Safety of gADNVs in vitro and in vivo

The cytotoxicity of gADNVs or Lips were evaluated against 4T1, B16F10, and MCF-10A cells by 3-(4,5-dimethyl-2-thiazolyl)-2,5-diphenyl-2-H-tetrazolium bromide (MTT) assay. Each cell line was seeded in 96-well plate with density of 10,000 cells per well. The gADNVs or Lips with the concentration of 0, 2 × 10^8^, 5 × 10^8^, 1 × 10^9^, 2 × 10^9^, or 3 × 10^9^ particles/mL were added after the cells adhered. Up to 24 h incubation, cells were washed twice with PBS and added with 100 μL MTT solution (1 mg/mL) for another 4 h incubation. Then, 150 μL DMSO was used to dissolve the resulting crystals after removing the MTT solution. After mixing for 10 min, the absorbance was measured by a microplate reader at the wavelength of 570 nm.

For the hemolysis assay of gADNVs, fresh blood was collected from mouse and mixed with 9 mL of PBS. Then the mixed solution was centrifuged under 1000×*g* for 5 min and washed thrice by PBS. The red blood cells (RBC) were separated and diluted with PBS to 2%. Lips and gADNVs with the same concentration (1.1 × 10^10^ particles/mL) were incubated with as-prepared RBC at 37 ℃ for 2 h, respectively. Then the final solutions were centrifuged, and the absorbances of supernatants were measured using a microplate reader at 540 nm. In addition, the as-prepared RBC solution treated with ultrapure-water was set as a positive control with the hemoglobin release rate of 100%, and the as-prepared RBC solution treated with PBS was set as a negative control. The hemolysis percentage was calculated as follows:

$$ {\text{Hemolysis percentage}} = \left[ {\left( {{\text{OD}}_{540}}\, {\text{of samples}} - {{\text{OD}}_{540}}\, {\text{of negative control}} \right)/\left( {{\text{OD}}_{540}}\, {\text{of positive control}} - {{\text{OD}}_{540}}\, {\text{of negative control}} \right)} \right] \times 100\% $$

To investigate the toxicity of gADNVs in vivo, mice were injected (intravenous injection) with gADNVs or Lips (1.1 × 10^10^ particles/mL) once per day for 7 days. After 24 h of the last injection, the serums of mice were collected for proinflammatory cytokines (TNF-α, IL-1β) analysis using enzyme-linked immunosorbent assay kits (Bioss Antibodies). Hematoxylin–eosin (H&E) staining of heart, liver, spleen, lung and kidney sections were also performed to evaluate the toxicity of gADNVs and Lips to organs.

### In vivo antitumor effect of ICG/gADNVs

The tumor bearing mouse model was built by subcutaneous injection of 5 × 10^6^ B16F10 cells at the right flank. Seven days post tumor inoculation, the tumor volume reached approximately 100 mm^3^. Mice were divided into seven groups (5 mice per group) randomly and received intratumor injection administration of PBS, freshly prepared groups (free ICG, ICG/Lips and ICG/gADNVs) and 30 days stored groups [ICG(+), ICG/Lips(+) and ICG/gADNVs(+)] (ICG/body weight of mouse, 0.5 mg/kg). After 1 h administration, mice were anesthetized and received 808 nm NR laser irradiation (1 W/cm^2^, 4 cycles, 5 min/cycle), the method for irradiating spot area calculation was described as shown in Additional file 1: Method and Materials S3. The tumor volume and mouse weight were recorded on alternate days. After 14 days treatment, tumors were separated and stored in tissue fixation fluid for 24 h for tissue sectioning and immunohistochemical staining.

### Statistical analysis

All results were analyzed at least three times and illustrated as mean ± standard deviation. Statistical significance was evaluated by using two-tail paired Student’s t-tests. *p* values < 0.05 were regarded as statistically significant (ns, no significance, *p < 0.05, **p < 0.01, ***p < 0.001, ****p < 0.0001).

## Supplementary Information

**Additional file 1: Table S1.** Viscosity analysis by ubbelohde viscometer. **Table S2.** Identification of proteins found in gADNV. **Table S3.** Identification of proteins found in rADNVs. **Table S4.** The full name of lipids mentioned in Fig. 2H. **Table S5.** The content of phytochemicals in ADNVs relative to their protein mass. **Fig. S1.** Schematic diagram of ubbelohde viscometer. **Fig. S2.** The total vesicle protein (μg) per gram tissue isolated obtained by centri-fugating for different times. **Fig. S3.** The analysis of aloeemodin (A), aloesin (B) and β-sitosterol (C) in gADNVs and rADNVs by HPLC. **Fig. S4.** A) The Lips with the mean size of 91.9 nm analyzed by NTA. Insert was the TEM micrograph of Lips. B) Characterization of gADNVs and Lips by Fourier transform infrared (FT-IR) spectroscopy. **Fig. S5.** Preparation and characterization of ICG/gADNVs and ICG/Lips. A) ICG loading efficiency analysis via changing the ratio of ICG and gADNVs. B) The particle size and morphology characterization of ICG/gADNVs by DLS and TEM. C) The particle size and morphology characterization of ICG/Lips by DLS and TEM. D) The zeta potential of ICG/gADNVs, ICG/Lips. Data in (A) and (D) represents mean values ± SD, n = 3. **Fig. S6.** A) Body weight changes of each group during phototherapy. B) Tumor volume changes of each group during therapy. C) H&E staining and TUNEL staining of tumor tissue in each group. Scale bar is 50 μm. D) Photos of mice showing the tumor growth at the first day and the 14th day under various conditions. Therein, ICG, ICG/Lips and ICG/gADNVs were the freshly prepare groups, ICG (+), ICG/Lips (+) and ICG/gADNVs (+) were the 30 days stored groups. Data in (B) represent mean values ± SD, n = 5. Statistical differences were analyzed by two-tailed student’s t-test. ****p < 0.0001.

## Data Availability

The datasets used and/or analyzed during the current study are available from the corresponding author on reasonable request.

## Abbreviations

gADNVs: Aloe gel derived nanovesicles
rADNVs: Aloe rind derived nanovesicles
ICG: Indocyanine green
ICG/gADNVs: GADNVs loaded with indocyanine green
EVs: Extracellular vesicles
ICG/Lips: Liposomes loaded with indocyanine green
PDNVs: Plants-derived nanovesicles
GDNVs: Grapefruit-derived nanovesicles
GDNs: Ginger derived nanovesicles
PA: Phosphatidic acid
PBS: Phosphate buffer solution
NTA: Nanoparticle tracking analysis
DLS: Dynamic light scattering
AFM: Micrographs by atomic force microscopy
TEM: Transmission electron microscopy
HPLC: High performance liquid chromatography
Lips: Liposomes
AAPH: 2,2-Azobis (2-amidinopropane) dihydrochloride
ORAC: Oxygen radical absorbance capacity assay
TX-100: Triton X-100
FBS: Fetal bovine serum
MTT: 3-(4,5-Dimethyl-2-thiazolyl)-2,5-diphenyl-2-H-tetrazolium bromide
H&E: Hematoxylin–eosin
PDI: Polydispersity index
TUNEL: Terminal deoxynucleotidyl transferase mediated nick end labeling
HSP 70: Heat shock protein
GAPDH: Glyceraldehyde-3-phosphate dehydrogenase
AGO: Argonaute
GlcCer: Glucosylceramide
Cer: Ceramide
PC: Phosphatidylcholine
FL: Fluorescein disodium
