# Supplementary material for: Aloe derived nanovesicle as a functional carrier for indocyanine green encapsulation and phototherapy
Source: J Nanobiotechnology. 2021 Dec 20;19:439. doi: 10.1186/s12951-021-01195-7 (PMC8686546; doi:10.1186/s12951-021-01195-7)
Supplement: Supplementary file 1 — Additional file 1: Table S1. Viscosity analysis by ubbelohde viscometer. Table S2. Identification of proteins found in gADNV. Table S3. Identification of proteins found in rADNVs. Table S4. The full name of lipids mentioned in Fig. 2H. Table S5. The content of phytochemicals in ADNVs relative to their protein mass. Fig. S1. Schematic diagram of ubbelohde viscometer. Fig. S2. The total vesicle protein (μg) per gram tissue isolated obtained by centri-fugating for different times. Fig. S3. The analysis of aloeemodin (A), aloesin (B) and β-sitosterol (C) in gADNVs and rADNVs by HPLC. Fig. S4. A) The Lips with the mean size of 91.9 nm analyzed by NTA. Insert was the TEM micrograph of Lips. B) Characterization of gADNVs and Lips by Fourier transform infrared (FT-IR) spectroscopy. Fig. S5. Preparation and characterization of ICG/gADNVs and ICG/Lips. A) ICG loading efficiency analysis via changing the ratio of ICG and gADNVs. B) The particle size and morphology characterization of ICG/gADNVs by DLS and TEM. C) The particle size and morphology characterization of ICG/Lips by DLS and TEM. D) The zeta potential of ICG/gADNVs, ICG/Lips. Data in (A) and (D) represents mean values ± SD, n = 3. Fig. S6. A) Body weight changes of each group during phototherapy. B) Tumor volume changes of each group during therapy. C) H&E staining and TUNEL staining of tumor tissue in each group. Scale bar is 50 μm. D) Photos of mice showing the tumor growth at the first day and the 14th day under various conditions. Therein, ICG, ICG/Lips and ICG/gADNVs were the freshly prepare groups, ICG (+), ICG/Lips (+) and ICG/gADNVs (+) were the 30 days stored groups. Data in (B) represent mean values ± SD, n = 5. Statistical differences were analyzed by two-tailed student’s t-test. ****p < 0.0001. [file 12951_2021_1195_MOESM1_ESM.docx]

**Additional information**

**Aloe derived nanovesicles as a functional carrier for indocyanine green encapsulation and phototherapy**

Lupeng Zeng ^a,b^, Huaying Wang ^a,b^, Wanhua Shi ^a,b^, Lingfan Chen ^c^, Tingting Chen ^a,b^, Guanyu Chen ^a,b^, Wenshen Wang ^e^, Jianming Lan ^a,b^, Zhihong Huang ^d^, Jing Zhang ^e^*, Jinghua Chen ^a,b^*

^a^ The School of Pharmacy, Fujian Medical University, Fuzhou, Fujian Province 350122 (P. R. China)

^b^ Fujian Key Laboratory of Drug Target Discovery and Structural and Functional Research, The School of Pharmacy, Fujian Medical University, Fuzhou, Fujian Province 350122 (P. R. China).

^c^ Fujian Province New Drug Safety Evaluation Centre, Fujian Medical University, Fuzhou, Fujian Province 350122, (P. R. China)

^d^ Public Technology Service Center, Fujian Medical University, Fuzhou, Fujian Province 350122 (P. R. China)

^e^ Department of Chemical Biology, College of Life Sciences, Fujian Agriculture and Forestry University, Fuzhou, Fujian Province 350002 (P. R. China)

* Corresponding authors:

Dr. Jinghua Chen, E-mail: [cjh_huaxue@126.com](mailto:cjh_huaxue@126.com).

Dr. Jing Zhang, E-mail: 517674177@qq.com.

Method and Materials

1. **Viscosity analysis of aloe juice**

Ubbelohde viscometer [1] was used for the viscosity (η) measurement of aloe juice. Briefly, the aloe tissue was mixed with PBS at different ratio (1:1, 1:2, 1:3, 1:4, 1:5, 1:6, w/v), and squeezed into aloe juice. 15 mL sample was added from tube A into viscometer, and blocked the tube C temporarily. Then the solution was pumped at tube B from ball F to 2/3 of ball G by a suction bulb. Subsequently, the tube C was opened, the timing started when the liquid level flowed through “a” scale and stopped at the “b” scale, measuring the time (T) required for the liquid between “a” and “b” to flow through the capillary. Water with a known viscosity at 25 ℃ (η_s_= 0.8937 mPa·S) was used as a reference for viscosity calculation, the time of water flow from “a” to “b” denoted as T_0_. The viscosity of aloe juice was calculated as follows:

Viscosity of aloe juice (η) = (η_s_ × T)/ T_0_

**
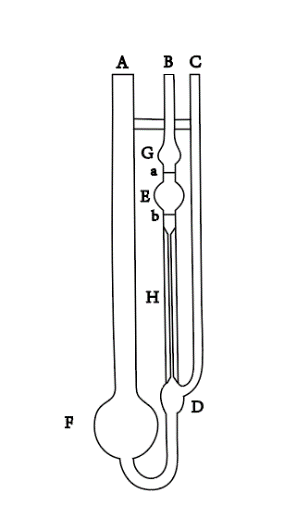
**

**Figure S1**. Schematic diagram of ubbelohde viscometer

**2. Preparation of Liposomes (Lips)**

The film hydration/extrusion method was performed for liposomes prepared [2]. DOPC: DOPE: DOPG had a mass ratio of 20:28:5 as the blank liposome formulation, dissolved in a compound solvent of chloroform and methanol (4:1, v/v) and vortexed completely. For preparation of lipid film, the mixed phospholipids solution in a 50 mL round-bottomed flask was placed on a rotary evaporator at 41 ℃ for 20 min. Subsequently, the organic solvent was removed and formed the lipid film preliminarily. To minimize the effect of organic solvent on liposomes formation, the residual solvent was removed in a vacuum desiccator overnight. Before extrusion, PBS (10 mM, 2 mL) as a hydrated solution was added in the flask to hydrate the lipid film into debris, followed by sonication for 15 min. In the end, the suspension solution was extruded with 0.2 μm and 0.1 μm polycarbonate-membrane (Avanti & Neverthirst) successively for 11 times extrusion producing a homogeneous liposomes solution.

**3.The calculation of irradiating spot area**

Based on the relevant report [3], the calculation of spot area is illustrated in Scheme S1. The diameter of optical fiber (d=400 μm) and the divergence angle (θ=25.2°) were fixed (provided by the manufacturer Beijing Niubite Science&Technology Co., Ltd.), thus the area of the spot depended only on the vertical distance (L) of irradiation. This could be expressed by Formula S1. In our study, the power of laser was 2 W and the vertical irradiation distance was 1.66 cm. Under these conditions, the spot area of irradiation was calculated as 2 cm^2^. The calculating method have been supplemented in the supporting information.


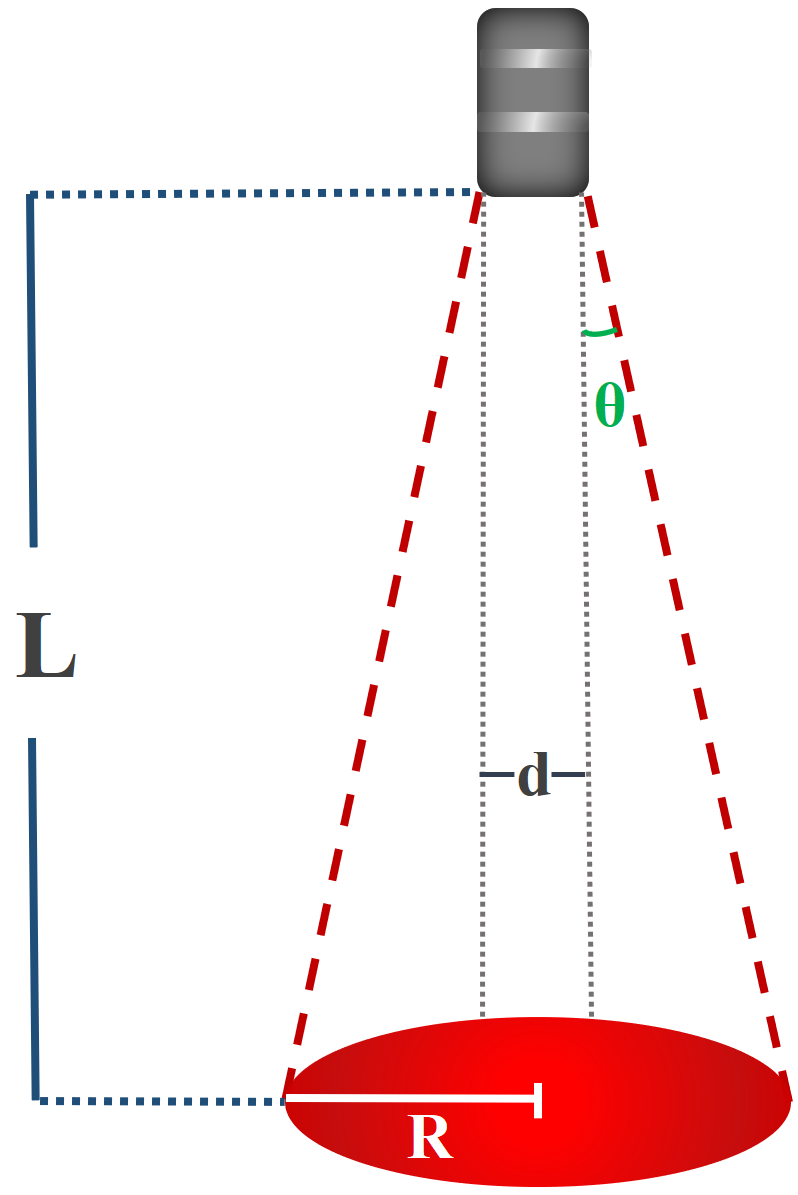


Scheme S1. Scheme of the optical fiber placement and the size of laser irradiation area.

Spot area = πR ²=π×[[d+2 L×tan(θ)]/2] ² ························· **Formula S1**

**Table S1** Viscosity analysis by ubbelohde viscometer

| **Viscosity analysis of aloe juice** | | |
| --- | --- | --- |
| Aloe tissue : PBS(w/v) | Aloe gel (mPa·S) | Aloe rind (mPa·S) |
| 1:1 | 4.65 | 2.02 |
| 1:2 | 3.77 | 1.65 |
| 1:3 | 2.98 | 1.57 |
| 1:4 | 2.04 | 1.53 |
| 1:5 | 1.65 | 1.49 |
| 1:6 | 1.61 | 1.46 |

**Table S2** Identification of proteins found in gADNVs

| **Identified proteins** | **Accession Number** | **Molecular Weight (kDa)** | **Isoelectric point (pI)** |
| --- | --- | --- | --- |
| **Aquaporin PIP1-3** | **Q08733** | **30.6** | **8.85** |
| **Phosphoglycerate kinase 2** | **P50318** | **49.9** | **8.27** |
| **Annexin D7** | **Q9LX07** | **36.5** | **6.9** |
| **Malate dehydrogenase 1** | **Q9ZP06** | **35.8** | **8.35** |
| **Phospholipase D alpha 1** | **Q38882** | **91.8** | **5.87** |
| **ABC transporter C family member 1** | **Q9C8G9** | **181.8** | **6.2** |
| **ABC transporter G family member 34** | **Q7PC80** | **164.2** | **6.92** |
| **Cytochrome b5** | **P49100** | **15.3** | **5.33** |
| **Syntaxin-71** | **Q9SF29** | **30** | **5.22** |
| **Fructose-bisphosphate aldolase 8** | **Q9LF98** | **38.5** | **6.46** |
| **UDP-glucuronic acid decarboxylase 5** | **Q9SN95** | **38.4** | **7.52** |
| **Membrane steroid-binding protein 1** | **Q9FVZ7** | **24.6** | **4.59** |
| **Elongation factor 1-alpha** | **P17786** | **49.3** | **9.06** |
| **Elongation factor 1-gamma 3** | **Q5Z627** | **47.4** | **6.47** |
| **Acyl-coenzyme A oxidase 2, peroxisomal** | **O65201** | **77.4** | **8.29** |
| **Acetyl-CoA carboxylase 1** | **Q8S6N5** | **252.7** | **6.4** |
| **Methylenetetrahydrofolate reductase 1** | **Q9SE94** | **66.4** | **5.91** |
| **Transmembrane 9 superfamily member 1** | **Q940G0** | **66.8** | **7.65** |
| **Glyceraldehyde-3-phosphate dehydrogenase GAPA1** | **P25856** | **42.5** | **7.75** |
| **Pyruvate decarboxylase 1** | **Q0DHF6** | **65.1** | **6.23** |
| **Dynamin-related protein 1A** | **P42697** | **68.1** | **8.31** |
| **Methylthioribose kinase** | **Q9C6D2** | **48** | **5.73** |
| **Peroxisomal membrane protein 11C** | **Q9LQ73** | **25.9** | **9.76** |
| **Citrate synthase 2** | **Q9LXS6** | **56.5** | **8.62** |
| **Sucrose synthase 1** | **P49040** | **92.9** | **6.2** |
| **Protein argonaute 10** | **Q9XGW1** | **110.8** | **9.23** |
| **ABC transporter G family member 40** | **Q9M9E1** | **160.9** | **8.06** |
| **Alpha-glucan phosphorylase 1** | **Q9LIB2** | **108.5** | **5.47** |
| **Lipid phosphate phosphatase gamma** | **Q6NLA5** | **25.7** | **7.96** |
| **Protein argonaute 9** | **Q84VQ0** | **100.5** | **9.06** |
| **Serine carboxypeptidase 3** | **P37891** | **55.4** | **6.14** |
| **Heat shock cognate protein 80** | **P36181** | **80.1** | **5.03** |
| **Sorbitol dehydrogenase** | **Q9FJ95** | **39.27** | **5.9** |
| Aquaporin PIP2-2 | Q6K215 | 30.4 | 7.81 |
| ATPase, plasma membrane-type | Q9SU58 | 105.7 | 6.52 |
| Heat shock protein70 | Q9C7X7 | 68.3 | 5.35 |
| Heat shock protein 90 | F4JFN3 | 90.5 | 5.39 |
| Glutathione S-transferase | Q8L7C9 | 25 | 5.78 |
| Malate dehydrogenase | Q08062 | 35.6 | 6.09 |
| ABC transporter F family member 4 | Q9M1H3 | 80.4 | 6.32 |
| 6-phosphogluconate dehydrogenas | Q9LI00 | 52.7 | 6.18 |
| Cytochrome f | Q1KXU6 | 35.2 | 8.57 |
| 14-3-3-like protein | P42643 | 29.9 | 4.81 |
| Isocitrate dehydrogenase | Q9SRZ6 | 45.7 | 6.57 |
| Elongation factor 2 | Q9ASR1 | 93.8 | 6.25 |
| Peroxisomal (S)-2-hydroxy-acid oxidase | Q10CE4 | 40.4 | 8.47 |
| Cysteine synthase | P47999 | 41.6 | 8.02 |
| Phosphoglycerate kinase | P12783 | 4.1 | 5.86 |
| Phosphoenolpyruvate carboxylase 1 | Q9MAH0 | 110.2 | 6.06 |
| Phosphoenolpyruvate carboxykinase (ATP) | Q9T074 | 73.4 | 7.08 |
| Actin-7 | P53492 | 16.8 | 4.27 |
| Actin-1 | Q10DV7 | 41.8 | 5.49 |
| Actin | P53500 | 42 | 5.49 |
| Aquaporin PIP1-2 | Q7XSQ9 | 30.7 | 8.63 |
| Enolase | P26300 | 47.8 | 5.95 |
| Adenosylhomocysteinase 1 | O23255 | 53.3 | 5.97 |
| Phospholipase D delta | Q9C5Y0 | 98.9 | 7.17 |
| ABC transporter B family member 25 | Q9LVM1 | 117.6 | 7.21 |
| ABC transporter G family member 36 | A2WSH0 | 162.8 | 7.25 |
| ABC transporter G family member 22 | Q93YS4 | 82.9 | 9.2 |
| Cytochrome c-1 | O23138 | 12.4 | 9.31 |
| Beta-glucosidase 1 | Q5QMT0 | 58 | 8.27 |
| Fructose-bisphosphate aldolase 2, | Q10A30 | 38.7 | 6.98 |
| Fructose-1,6-bisphosphatase 1 | P25851 | 45.1 | 5.4 |
| Heat shock protein 81 | A2YWQ | 80.1 | 5.07 |
| Transmembrane 9 superfamily member 7 | Q9LIC2 | 74.2 | 8.5 |
| Transmembrane 9 superfamily member 3 | Q9ZPS7 | 68 | 7.08 |
| Transmembrane 9 superfamily member 11 | Q9FYQ8 | 74.5 | 7.96 |
| S-adenosylmethionine synthase | Q0DKY4 | 43.2 | 6.14 |
| UDP-glucose 6-dehydrogenase 4 | P57751 | 51.9 | 6.01 |
| UDP-glucose 6-dehydrogenase 3 | Q9LF33 | 53.1 | 6.04 |
| UDP-glucose 4-epimerase 2 | Q9T0A7 | 38.4 | 6.73 |
| Vesicle transport protein | Q94AU2 | 25.3 | 9 |
| Phosphomannomutase | Q7XPW5 | 28.2 | 5.83 |
| Vesicle-fusing ATPase | Q9M0Y8 | 81.4 | 6.1 |
| Histone H2B.10 | A2WKS3 | 16.5 | 10.02 |
| Elongation factor 1-delta | P48006 | 25.1 | 4.56 |
| Glyceraldehyde-3-phosphate dehydrogenase | Q43247 | 36.4 | 7.47 |
| Glyceraldehyde-3-phosphate dehydrogenase GAPC2 | Q9FX54 | 36.9 | 7.18 |
| UTP--glucose-1-phosphate uridylyltransferase 2 | Q9M9P3 | 51.7 | 6.13 |
| Serine hydroxymethyltransferase 4 | O23254 | 51.7 | 7.23 |
| Hypersensitive-induced response protein 1 | Q6ZIV7 | 31.4 | 5.35 |
| Hypersensitive-induced response protein 4 | Q9FHM7 | 32.4 | 5.43 |
| Pyruvate kinase | Q42806 | 55.3 | 7.56 |
| Pyruvate decarboxylase 3 | Q0D3D2 | 62.6 | 5.95 |
| Glutamine synthetase cytosolic isozyme | Q9LVI8 | 38.6 | 6.06 |
| Peroxisomal isocitrate dehydrogenase [NADP] | Q9SLK0 | 47.2 | 7.71 |
| Lactoylglutathione lyase | Q948T6 | 32.5 | 5.67 |
| Mannose-1-phosphate guanylyltransferase 1 | O22287 | 39.6 | 6.73 |
| Peroxiredoxin-2B | Q9XEX2 | 17.4 | 5.36 |
| ATP-dependent 6-phosphofructokinase 1 | Q9M0F9 | 52 | 7.55 |
| Adenine phosphoribosyltransferase 3 | Q9SUW2 | 20.3 | 6.09 |
| L-ascorbate peroxidase | Q05431 | 27.5 | 6.13 |
| Dynamin-related protein 1E | Q9FNX5 | 69.8 | 7.52 |
| Alcohol dehydrogenase 2 | Q0ITW7 | 41.2 | 6.46 |
| Sucrose synthase 2 | P30298 | 92.1 | 6.39 |
| Adenosine kinase 2 | Q9LZG0 | 37.8 | 5.26 |
| Phosphopantothenate--cysteine ligase 1 | Q69S81 | 35.3 | 8.03 |
| Mitogen-activated protein kinase 6 | Q39026 | 45 | 5.48 |
| Phosphoinositide phospholipase C 7 | Q9LY51 | 66.4 | 5.9 |
| 5-Methyltetrahydropteroyltriglutamate--homocysteine methyltransferase 2 | Q9SRV5 | 84.5 | 6.51 |
| Phosphopantothenoylcysteine decarboxylase | Q69K55 | 24.1 | 7.34 |
| Catalase | O48561 | 56.7 | 7.28 |
| Catalase isozyme A | Q0E4K1 | 56.7 | 7.01 |
| Malate dehydrogenase | Q42972 | 37.4 | 7.88 |
| Aldehyde dehydrogenase family 2 member B4 | Q9SU63 | 58.6 | 7.46 |
| Glucose-6-phosphate 1-dehydrogenase 6 | Q9FJI5 | 59.1 | 6.43 |
| Glucose-6-phosphate isomerase | P42862 | 62.5 | 7.28 |
| Phosphoinositide phospholipase C 2 | Q39033 | 66.1 | 6.54 |
| Adenylate kinase 3 | Q08479 | 26.4 | 8.28 |
| Fructokinase-1 | Q0JGZ6 | 34.7 | 5.19 |
| 6-phosphogluconate dehydrogenase | Q9FWA3 | 53.5 | 7.42 |
| Aconitate hydratase 1 | Q42560 | 98.1 | 6.4 |
| GDP-mannose 3,5-epimerase | Q93VR3 | 42.7 | 6.21 |
| Glycolipid transfer protein 1 | O22797 | 22.7 | 7.39 |
| 3-ketoacyl-CoA thiolase 5, peroxisomal | Q570C8 | 47.9 | 7.4 |
| Citrate synthase 3, peroxisomal | Q9SJH7 | 38.6 | 6.05 |
| Methylthioribose-1-phosphate isomerase | B6TZD1 | 56.1 | 7.66 |
| Phosphoacetylglucosamine mutase | Q6ZDQ1 | 60.3 | 5.45 |
| Vesicle-associated membrane protein 713 | Q9LFP1 | 25.3 | 9.06 |
| Methylenetetrahydrofolate reductase 1 | Q9SE60 | 66.2 | 5.82 |
| 1-aminocyclopropane-1-carboxylate oxidase 1 | A2Z1W9 | 36.4 | 5.15 |
| Protein transport protein SEC13 homolog A | Q9SRI1 | 32.6 | 6.05 |
| Calcium-dependent lipid-binding protein | Q9LEX1 | 55.1 | 8.21 |
| Osmotin-like protein | Q41350 | 27.2 | 7.88 |
| Protein argonaute 1B | Q7XSA2 | 123.5 | 9.5 |
| Protein argonaute 1D | Q5Z5B2 | 115.9 | 9.03 |
| Transportin-1 | Q8H0U4 | 99 | 4.94 |
| Sterol 14-demethylas | Q9SAA9 | 55.5 | 8.29 |
| Sterol 3-beta-glucosyltransferase UGT80A2 | Q9M8Z7 | 69.2 | 6.74 |
| Transportin-1 | Q8H0U4 | 99 | 4.94 |
| Sterol 14-demethylas | Q9SAA9 | 55.5 | 8.29 |
| Sterol 3-beta-glucosyltransferase UGT80A2 | Q9M8Z7 | 69.2 | 6.74 |
| Glycolipid transfer protein 1 | O22797 | 22.7 | 7.39 |
| Homocysteine S-methyltransferase 2 | Q9M1W4 | 36.4 | 5.16 |

Note: The proteins colored in red and bold were the same proteins in both gADNVs and rADNVs.

**Table S3** Identification of proteins found in rADNVs

| **Identified proteins** | **Accession Number** | **Molecular Weight (kDa)** | **Isoelectric point (pI)** |
| --- | --- | --- | --- |
| **Aquaporin PIP1-3** | **Q08733** | **30.6** | **8.85** |
| **Phosphoglycerate kinase 2** | **P50318** | **49.9** | **8.27** |
| **Annexin D7** | **Q9LX07** | **36.5** | **6.9** |
| **Malate dehydrogenase 1** | **Q9ZP06** | **35.8** | **8.35** |
| **Phospholipase D alpha 1** | **Q38882** | **91.8** | **5.87** |
| **ABC transporter C family member 1** | **Q9C8G9** | **181.8** | **6.2** |
| **ABC transporter G family member 34** | **Q7PC80** | **164.2** | **6.92** |
| **Cytochrome b5** | **P49100** | **15.3** | **5.33** |
| **Syntaxin-71** | **Q9SF29** | **30** | **5.22** |
| **Fructose-bisphosphate aldolase 8,** | **Q9LF98** | **38.5** | **6.46** |
| **UDP-glucuronic acid decarboxylase 5** | **Q9SN95** | **38.4** | **7.52** |
| **Membrane steroid-binding protein 1** | **Q9FVZ7** | **24.6** | **4.59** |
| **Elongation factor 1-alpha** | **P17786** | **49.3** | **9.11** |
| **Elongation factor 1-gamma 3** | **Q5Z627** | **47.4** | **6.47** |
| **Acyl-coenzyme A oxidase 2** | **O65201** | **77.4** | **8.29** |
| **Acetyl-CoA carboxylase 1** | **Q8S6N5** | **252.7** | **6.4** |
| **Methylenetetrahydrofolate reductase 1** | **Q9SE94** | **66.4** | **5.91** |
| **Transmembrane 9 superfamily member 1** | **Q940G0** | **66.8** | **7.65** |
| **Glyceraldehyde-3-phosphate dehydrogenase GAPA1** | **P25856** | **42.5** | **7.75** |
| **Pyruvate decarboxylase 1** | **Q0DHF6** | **65.1** | **6.23** |
| **Dynamin-related protein 1A** | **P42697** | **68.1** | **8.31** |
| **Methylthioribose kinase** | **Q9C6D2** | **48** | **5.73** |
| **Peroxisomal membrane protein 11C** | **Q9LQ73** | **25.9** | **9.76** |
| **Citrate synthase 2** | **Q9LXS6** | **56.6** | **8.62** |
| **Sucrose synthase 1** | **P49040** | **92.9** | **6.2** |
| **Protein argonaute 10** | **Q9XGW1** | **110.8** | **9.23** |
| **ABC transporter G family member 40** | **Q9M9E1** | **160.9** | **8.06** |
| **Alpha-glucan phosphorylase 1** | **Q9LIB2** | **108.5** | **5.47** |
| **Lipid phosphate phosphatase gamma** | **Q6NLA5** | **25.7** | **7.96** |
| **Protein argonaute 9** | **Q84VQ0** | **100.5** | **9.06** |
| **Serine carboxypeptidase 3** | **P37891** | **55.4** | **6.14** |
| **Heat shock cognate protein 80** | **P36181** | **80.1** | **5.03** |
| **Sorbitol dehydrogenase** | **Q9FJ95** | **39.2** | **5.97** |
| Aquaporin PIP2-2 | P43287 | 30.4 | 7.81 |
| Plasma membrane ATPase | Q7XPY2 | 104.8 | 6.79 |
| Heat shock 70 kDa protein | Q6Z7B0 | 73.3 | 5.19 |
| Heat shock protein 81 | A2YWQ1 | 80.1 | 5.07 |
| Heat shock protein 90 | P27323 | 80.6 | 5.05 |
| Glutathione S-transferase U24 | Q9SHH6 | 25.3 | 6.16 |
| Malate dehydrogenase [NADP] | Q8H1E2 | 48.3 | 6.16 |
| ABC transporter C family member 4 | Q7DM58 | 169 | 7.65 |
| 6-phosphogluconate dehydrogenase | Q9FWA3 | 53.5 | 7.42 |
| Cytochrome f | Q0G9U9 | 35.3 | 8.78 |
| 14-3-3 like protein | P93214 | 29.4 | 4.82 |
| Isocitrate dehydrogenase [NADP] | Q06197 | 46 | 6.23 |
| Elongation factor 1-alpha | P17786 | 49.3 | 9.11 |
| Elongation factor 1-gamma 3 | Q5Z627 | 47.4 | 6.47 |
| Elongation factor 1-alpha | P17786 | 49.3 | 9.11 |
| Elongation factor 1-gamma 3 | Q5Z627 | 47.4 | 6.47 |
| (S)-2-hydroxy-acid oxidase | Q9LRS0 | 40.3 | 8.97 |
| Cysteine synthase | Q9XEA8 | 34.3 | 5.41 |
| Histone H2B.2 | O65818 | 15.4 | 10.08 |
| Glutathione S-transferase | Q8L7C9 | 25 | 5.78 |
| Probable histone H2A.1 | A2YMC5 | 14 | 10.05 |
| Putative actin-5 | Q8RYC2 | 42.1 | 5.33 |
| Phosphoglycerate kinase 3 | Q9SAJ4 | 42.1 | 5.68 |
| Alcohol dehydrogenase 1 | Q75ZX4 | 41 | 6.65 |
| Alcohol dehydrogenase class-3 | Q0DWH1 | 40.8 | 7.17 |
| Malate dehydrogenase 2 | Q9ZP05 | 37.3 | 7.99 |
| ABC transporter B family member 28 | Q8LPQ6 | 77.9 | 8.98 |
| ABC transporter C family member 2 | Q42093 | 182 | 6.42 |
| ABC transporter E family member 2 | Q8LPJ4 | 68.3 | 7.96 |
| ABC transporter G family member 7 | Q9ZU35 | 78.9 | 5.94 |
| Glucose-6-phosphate 1-dehydrogenase 2 | Q9FY99 | 67.1 | 8.29 |
| Glucose-6-phosphate 1-dehydrogenase 1 | Q43727 | 65.4 | 7.74 |
| Cytochrome b6-f complex iron-sulfur subunit | Q69S39 | 23.9 | 8.25 |
| Cytochrome c oxidase subunit 6b-1 | Q9S7L9 | 21.2 | 4.34 |
| Cytochrome b6 | Q0G9T2 | 24.1 | 9.03 |
| Cytochrome b559 subunit beta | P60125 | 4.5 | 10.74 |
| Superoxide dismutase 3 | Q9FK60 | 16.9 | 7.64 |
| Fructose-bisphosphate aldolase 3 | Q9ZU52 | 42.3 | 8.09 |
| Transmembrane 9 superfamily member 12 | F4JRE0 | 74.1 | 6.18 |
| Isocitrate dehydrogenase [NAD] regulatory subunit 3 | O81796 | 39.9 | 7.44 |
| Acetyl-CoA carboxylase 2 | F4I1L3 | 262.6 | 7.09 |
| Elongation factor 1-delta 1 | P48006 | 25.1 | 4.56 |
| Universal stress protein | Q8VYN9 | 26.2 | 6.57 |
| Calcineurin B-like protein 3 | Q8LEM7 | 26 | 4.93 |
| Formate--tetrahydrofolate ligase | Q9SPK5 | 67.8 | 6.71 |
| L-ascorbate peroxidase S | Q42592 | 40.4 | 8.28 |
| Bifunctional enolase 2/transcriptional activator | P25696 | 47.7 | 5.77 |
| 1,4-alpha-glucan-branching enzyme 2-1 | O23647 | 97.6 | 5.62 |
| Ubiquitin-activating enzyme E1 1 | P93028 | 120.2 | 5.27 |
| Nucleoside diphosphate kinase 1 | P39207 | 16.5 | 6.79 |
| Protein argonaute 1 | Q6EU14 | 120.4 | 9.39 |
| Protein argonaute 4 | Q9SDG8 | 100.6 | 8.97 |
| Serine/threonine-protein kinase SAPK10 | Q75H77 | 40.7 | 4.97 |
| Calmodulin | P27161 | 16.9 | 4.27 |
| V-type proton ATPase subunit E1 | Q39258 | 26 | 6.4 |
| UDP-sugar pyrophosphorylase | Q5Z8Y4 | 67.5 | 6.87 |
| Membrane-associated protein | O80796 | 36.4 | 9.11 |
| Protein transport protein Sec24-like CEF | Q9M291 | 117.6 | 7.21 |
| Thioredoxin reductase NTRC | Q70G58 | 56.1 | 6.49 |
| Xylose isomerase | Q9FKK7 | 53.7 | 5.87 |
| Aspartic proteinase A3 | Q9XEC4 | 55.5 | 7.14 |
| Dynamin-related protein 1C | Q8LF21 | 68.7 | 7.56 |
| Urease accessory protein G | O64700 | 30.1 | 6.52 |
| Sucrose synthase 4 | Q10LP5 | 92.2 | 6.54 |
| ATP synthase subunit b | P48186 | 21 | 9.23 |
| Pre-mRNA-processing-splicing factor 8A | Q9SSD2 | 275.3 | 8.79 |
| Hypersensitive-induced response protein 3 | Q9SRH6 | 31.3 | 5.85 |
| Triosephosphate isomerase | P12863 | 27 | 5.68 |
| Mitogen-activated protein kinase 1 | Q84UI5 | 44.8 | 5.74 |
| Isocitrate dehydrogenase [NAD] regulatory subunit 3 | O81796 | 39.9 | 7.44 |
| Aldehyde dehydrogenase family 3 member I1 | Q8W033 | 60.1 | 8.51 |
| Glyceraldehyde-3-phosphate dehydrogenase GAPB | P25857 | 47.6 | 6.8 |
| Glyceraldehyde-3-phosphate dehydrogenase 3 | Q43247 | 36.4 | 7.47 |
| Glycine dehydrogenase (decarboxylating) 2 | O80988 | 113.7 | 6.65 |
| 25.3 kDa vesicle transport protein | Q94AU2 | 25.3 | 9 |
| Uridine 5'-monophosphate synthase | Q8RZA1 | 50.8 | 6.71 |
| Beta-adaptin-like protein B | Q9SUS3 | 99.3 | 5.05 |
| Pyruvate, phosphate dikinase 2 | Q42368 | 96 | 5.8 |
| Serine--glyoxylate aminotransferase | Q56YA5 | 44.2 | 7.83 |
| Glutamate decarboxylase 2 | Q42472 | 56.1 | 5.4 |
| Cysteine synthase 1 | P47998 | 33.8 | 6.14 |
| Leucine aminopeptidase | Q9FY49 | 69.2 | 5.24 |
| Methylthioribose kinase 1 | Q7XR61 | 48.4 | 6.54 |
| Mitogen-activated protein kinase 4 | Q39024 | 42.8 | 6.11 |
| Non-specific phospholipase C4 | Q9SRQ7 | 60.7 | 6.23 |
| Ricin B-like lectin R40G3 | Q6Z4N4 | 22.8 | 7.81 |
| Glutathione S-transferase | Q9SHH7 | 25.6 | 5.3 |
| Glutamine synthetase | P04771 | 39.2 | 5.6 |
| Sucrose-phosphate synthase 1 | Q94BT0 | 117.2 | 6.43 |
| Alpha-glucan water dikinase 1 | Q9SAC6 | 156.5 | 5.92 |
| Phosphoglucan | Q6ZY51 | 131.2 | 6.24 |
| Phosphoinositide phosphatase SAC6 | Q7X911 | 67.6 | 7.75 |
| Phosphoglucan phosphatase DSP4 | Q9FEB5 | 42.6 | 6.55 |
| Starch synthase 3 | F4IAG2 | 118.4 | 6.52 |
| Phytochrome B1 | Q9ZS62 | 125.5 | 6.15 |
| Adenylate kinase 4 | Q08480 | 26.7 | 7.8 |

Note: The proteins colored in red and bold were the same proteins in both gADNVs and rADNVs.

**Table S4.** The full name of lipids mentioned in Figure 2H

| DGTS | Diacylglyceryltrimethylhomoserine |
| --- | --- |
| GlcADG | Glucuronosyldiacylglycerol |
| AcylGlcADG | AcylGlucuronosyldiacylglycerol |
| PEtOH | Phosphatidylethanol |
| PMeOH | Phosphatidylmethanol |
| HBMP | Hemibismonoacylglycerophosphate |
| SQDG | Sulfoquinovosyldiacylglycerol |
| DGDG | Digalactosyldiacylglycerol |
| MGDG | Monodigalactosyldiacylglycerol |
| TAG | Triacylglycerols |
| DAG | Ditriacylglycerols |
| GlcCer | Glucocerebroside |
| Cer | Ceramide |
| SM | Sphingomyelins |
| CL | Cardiolipins |
| PA | Phosphatidic acid |
| PG | Phosphatidyl glycerol |
| PI | Phosphatidyl inositol |
| PS | Phosphatidylserine |
| PE | Phosphatidyl ethanolamine |
| PC | Phosphatidyl choline |

| **Phytochemicals/ADNVs（μg/mg）** | **Aloe-emodin** | **Aloesin** | **β-sitosterol** |
| --- | --- | --- | --- |
| rADNVs | 2.264 | 0.651 | 100.702 |
| gADNVs | 0.187 | 0.627 | 275.561 |

**Table S5.** The content of phytochemicals in ADNVs relative to their protein mass

**Figure S2.** The total vesicle protein (μg) per gram tissue isolated obtained by centri-fugating for different times.


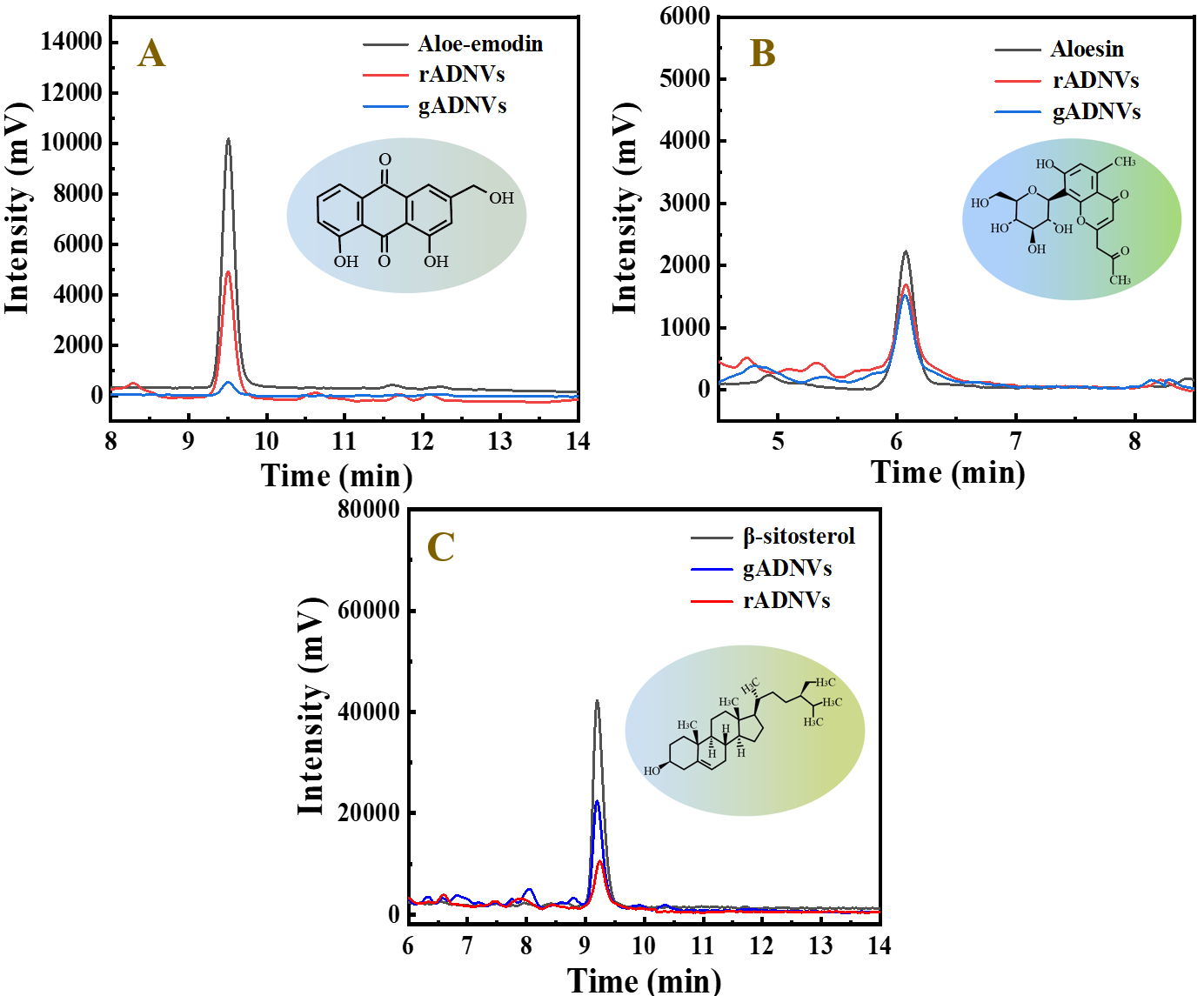


**Figure S3.** The analysis of aloe-emodin (A), aloesin (B) and β-sitosterol (C) in gADNVs and rADNVs by HPLC


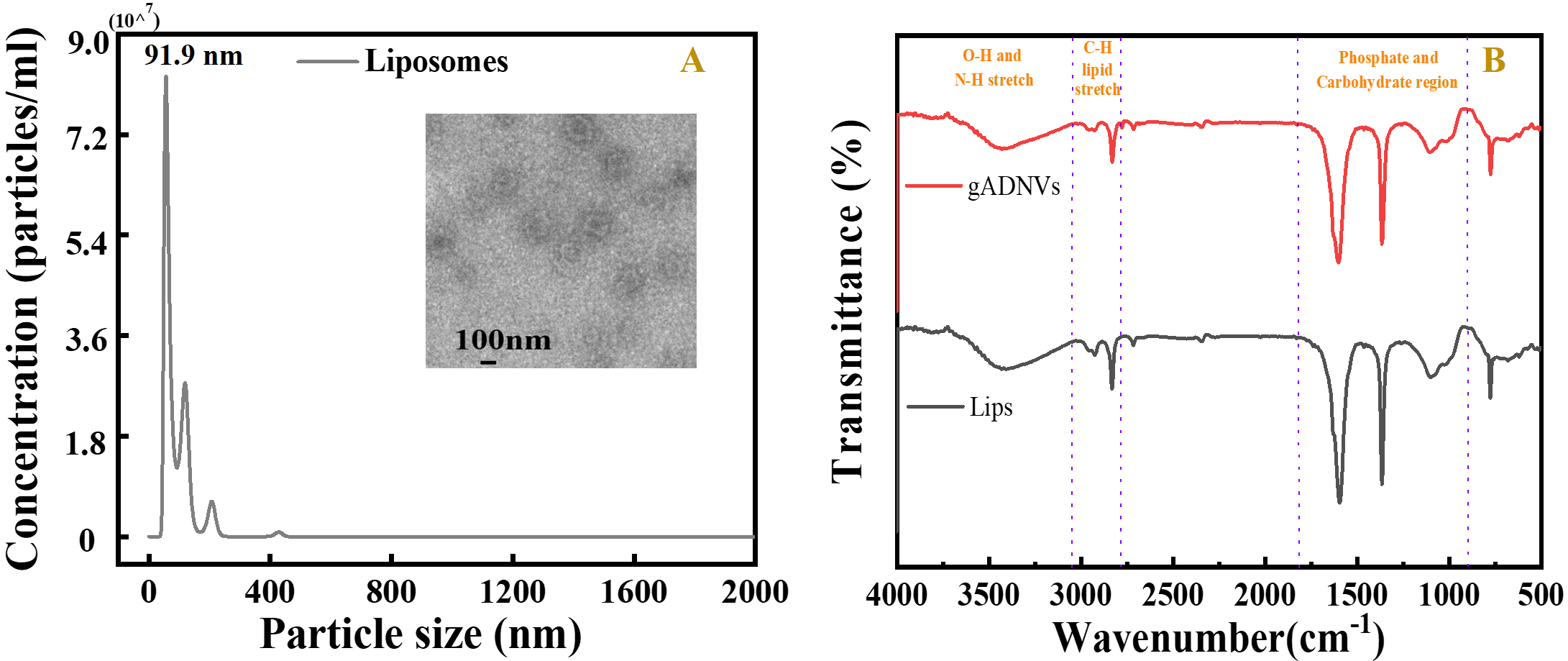


**Figure S4.** A) The Lips with the mean size of 91.9 nm analyzed by NTA. Insert was the TEM micrograph of Lips. B) Characterization of gADNVs and Lips by Fourier transform-infrared (FT-IR) spectroscopy.


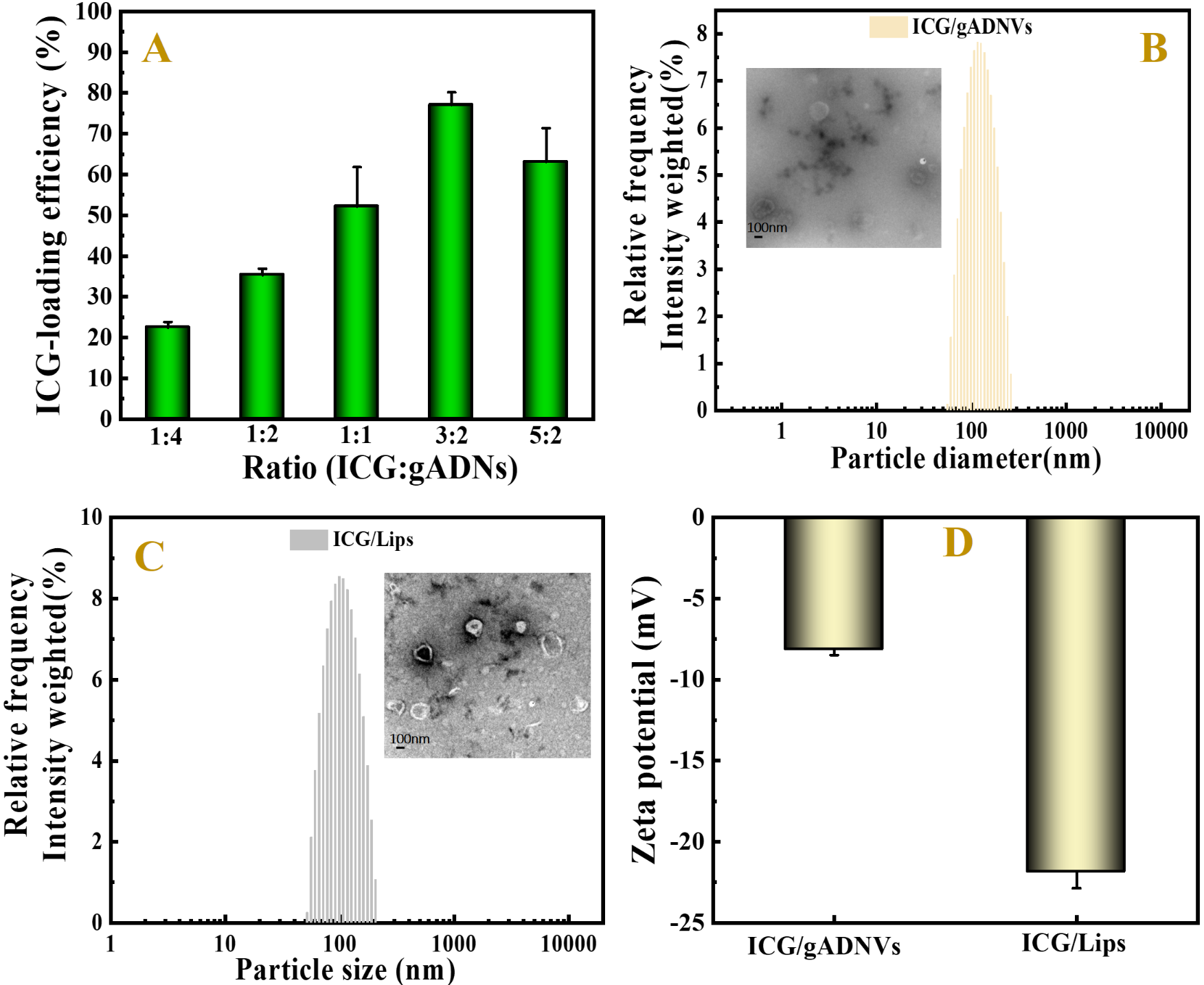


**Figure S5.** Preparation and characterization of ICG/gADNVs and ICG/Lips. A) ICG loading efficiency analysis via changing the ratio of ICG and gADNVs. B) The particle size and morphology characterization of ICG/gADNVs by DLS and TEM. C) The particle size and morphology characterization of ICG/Lips by DLS and TEM. D) The zeta potential of ICG/gADNVs, ICG/Lips. Data in (A) and (D) represents mean values ± SD, n=3.


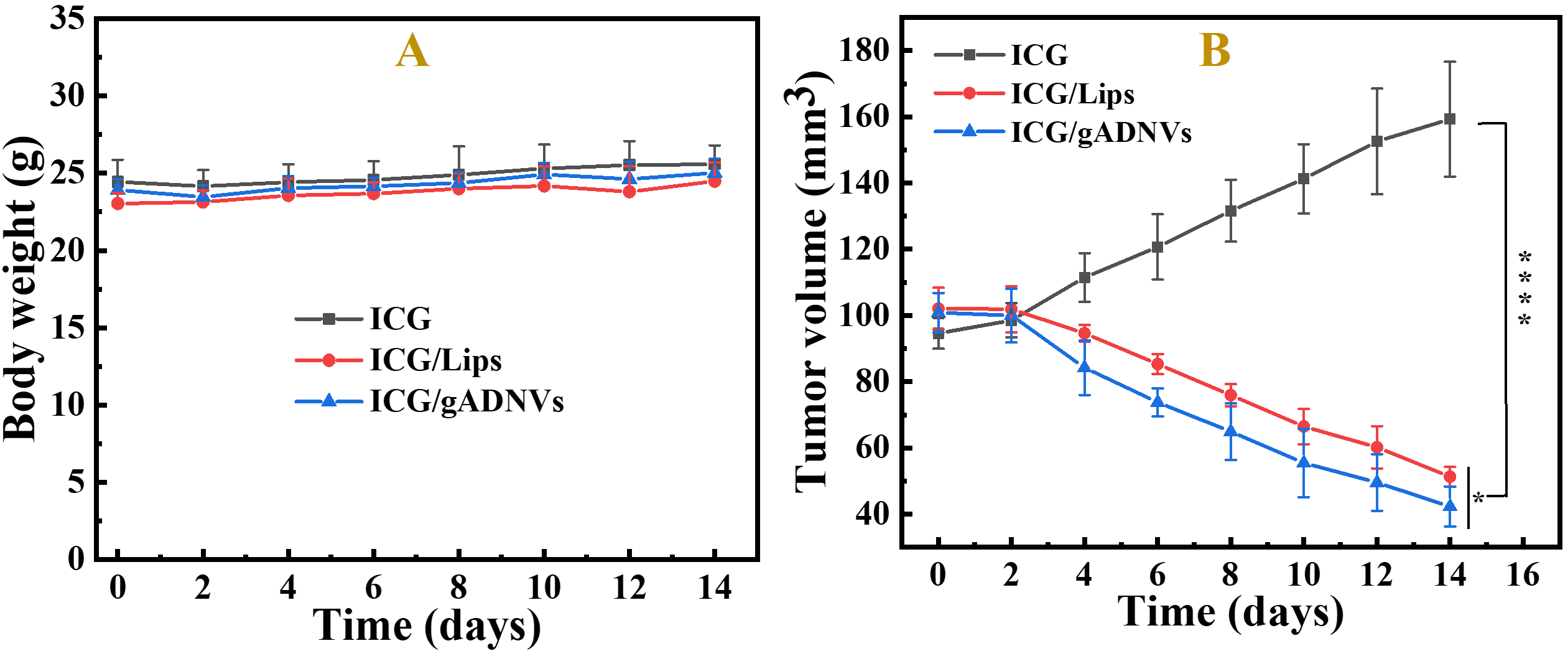


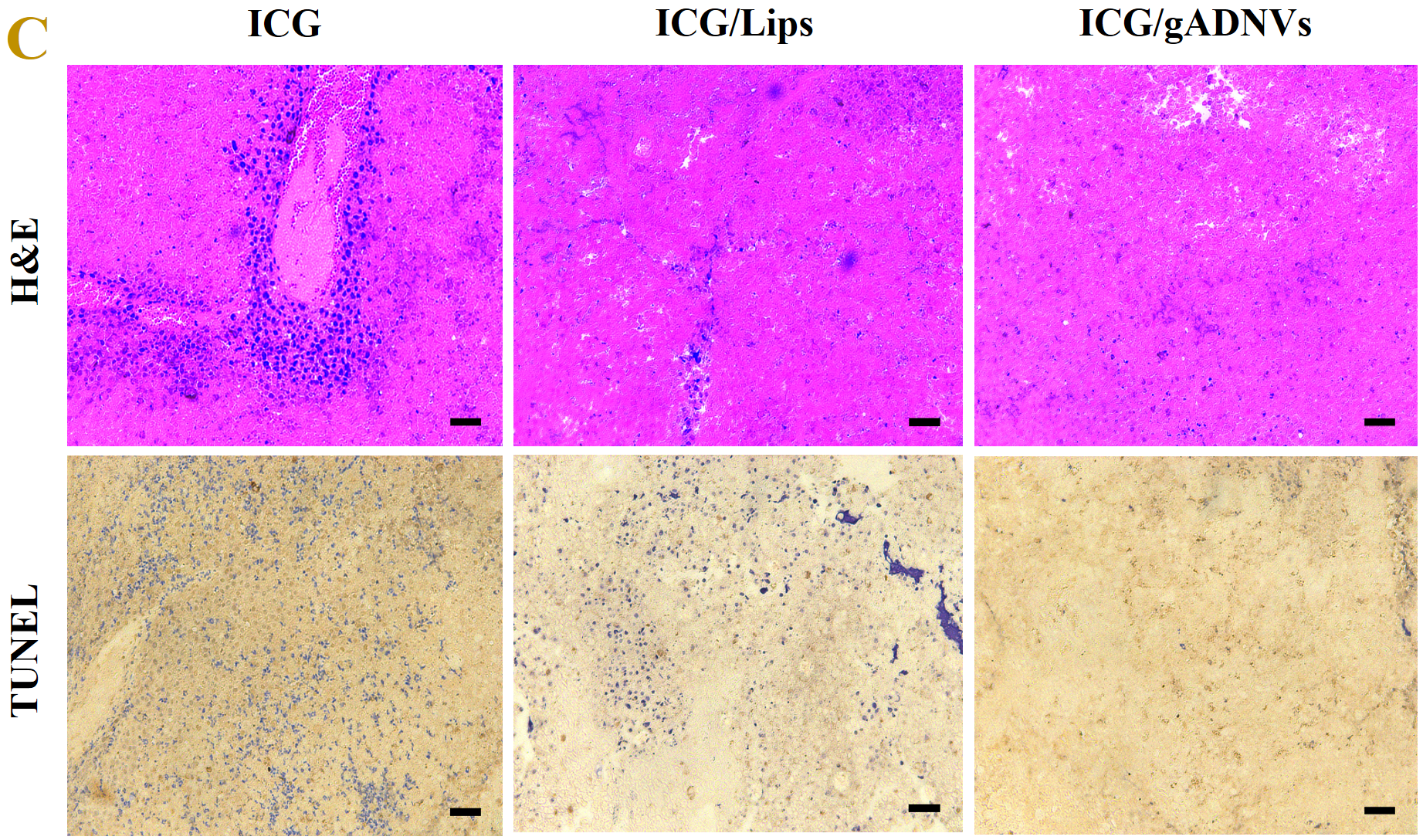


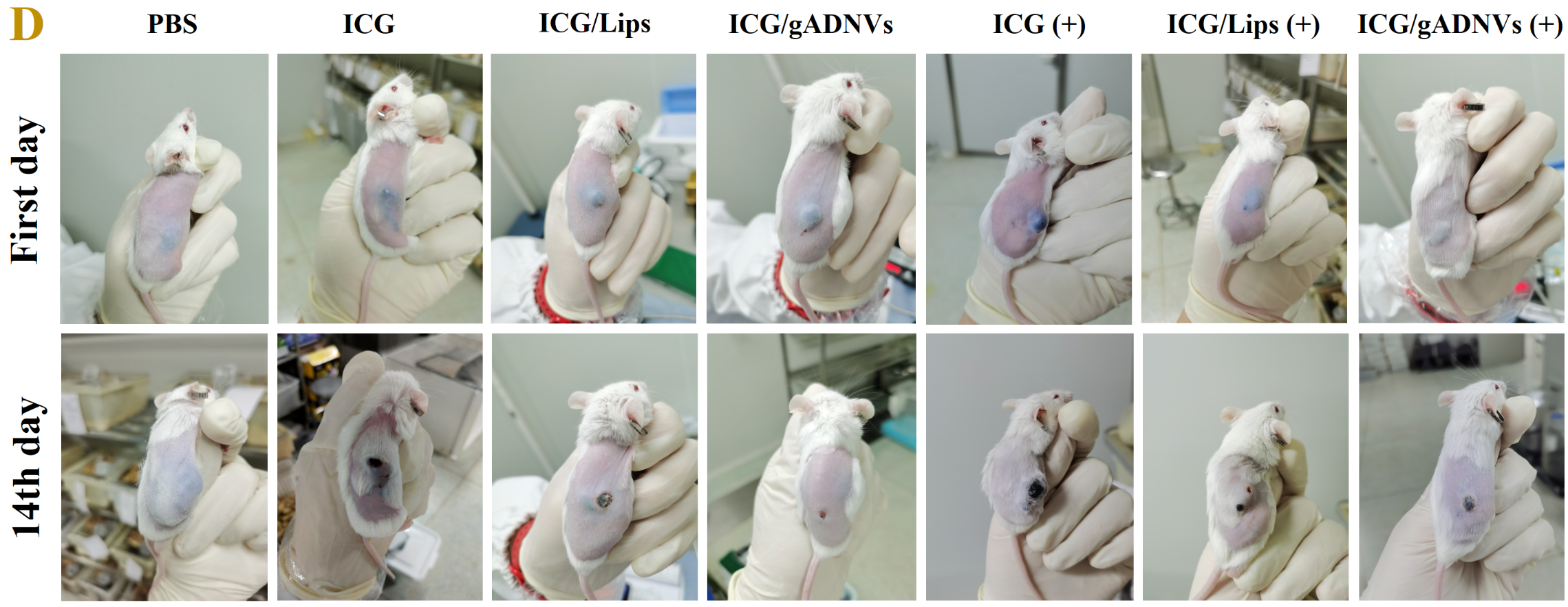


**Figure S6.** A) Body weight changes of each group during phototherapy. B) Tumor volume changes of each group during therapy. C) H&E staining and TUNEL staining of tumor tissue in each group. Scale bar is 50 μm. D) Photos of mice showing the tumor growth at the first day and the 14th day under various conditions. Therein, ICG, ICG/Lips and ICG/gADNVs were the freshly prepare groups, ICG (+), ICG/Lips (+) and ICG/gADNVs (+) were the 30 days stored groups. Data in (B) represent mean values ± SD, n=5. Statistical differences were analyzed by two-tailed student’s t-test. *****p* < 0.0001.

**References**

[1] Gu H, Tang X, Hong RY, Feng W G, Xie HD, Chen D X, Badami D. Ubbelohde viscometer measurement of water-based Fe3O4 magnetic fluid prepared by coprecipitation. J Magn Magn Mater. 2013; 348: 88-92.

[2] Lajunen T, Nurmi R, Wilbie D, Ruoslahti T, Johansson NG, Korhonen O, Rog T, Bunker A, Ruponen M, Urtti A. The effect of light sensitizer localization on the stability of indocyanine green liposomes. J Control Release. 2018; 284: 213-223.

[3] Sun J, Song L, Fan Y, Tian L, Luan S, Niu S, Ren L, Ming W, Zhao J. Synergistic photodynamic and photothermal antibacterial nanocomposite membrane triggered by single NIR light source. ACS Appl Mater interfaces. 2019; 11(30): 26581-26589.
